# Exogenous application of nanocarrier‐mediated double‐stranded RNA manipulates physiological traits and defence response against bacterial diseases

**DOI:** 10.1111/mpp.13417

**Published:** 2024-01-19

**Authors:** Garima Pal, Kishor D. Ingole, Prabhu Srinivas Yavvari, Priyanka Verma, Ankit Kumari, Chetan Chauhan, Darshna Chaudhary, Aasheesh Srivastava, Avinash Bajaj, Ramu S. Vemanna

**Affiliations:** ^1^ Laboratory of Plant Functional Genomics Regional Centre for Biotechnology, NCR Biotech Science Cluster Faridabad India; ^2^ Department of Chemistry Indian Institute of Science Education and Research Bhopal India; ^3^ Laboratory of Nanotechnology and Chemical Biology Regional Centre for Biotechnology, NCR Biotech Science Cluster Faridabad India; ^4^ Plant Genetic Engineering Lab Centre for Biotechnology, Maharshi Dayananda University Rohtak India

**Keywords:** biomass, crop protection, dsRNA, plant endogenous genes, polymer, RNAi, *Xanthomonas*

## Abstract

Stability and delivery are major challenges associated with exogenous double‐stranded RNA (dsRNA) application into plants. We report the encapsulation and delivery of dsRNA in cationic poly‐aspartic acid‐derived polymer (CPP6) into plant cells. CPP6 stabilizes the dsRNAs during long exposure at varied temperatures and pH, and protects against RNase A degradation. CPP6 helps dsRNA uptake through roots or foliar spray and facilitates systemic movement to induce endogenous gene silencing. The fluorescence of *Arabidopsis GFP‐*overexpressing transgenic plants was significantly reduced after infiltration with *gfp*‐dsRNA‐CPP6 by silencing of the transgene compared to plants treated only with *gfp*‐dsRNA. The plant endogenous genes *flowering locus T* (*FT*) and *phytochrome interacting factor 4* (*PIF4*) were downregulated by a foliar spray of *ft*‐dsRNA‐CPP6 and *pif4*‐dsRNA‐CPP6 in *Arabidopsis*, with delayed flowering and enhanced biomass. The rice *PDS* gene targeted by *pds*‐dsRNA‐CPP6 through root uptake was effectively silenced and plants showed a dwarf and albino phenotype. The NaCl‐induced *OsbZIP23* was targeted through root uptake of *bzip23*‐dsRNA‐CPP6 and showed reduced transcripts and seedling growth compared to treatment with naked dsRNA. The negative regulators of plant defence *SDIR1* and *SWEET14* were targeted through foliar spray to provide durable resistance against bacterial leaf blight disease caused by *Xanthomonas oryzae* pv. *oryzae* (Xoo). Overall, the study demonstrates that transient silencing of plant endogenous genes using polymer‐encapsulated dsRNA provides prolonged and durable resistance against Xoo, which could be a promising tool for crop protection and for sustaining productivity.

## Graphical abstract

Efficient targeting of negative regulators of plant growth and disease susceptibility genes by nano carrier‐mediated dsRNAs can improve agronomic traits and crop protection against bacterial pathogens.

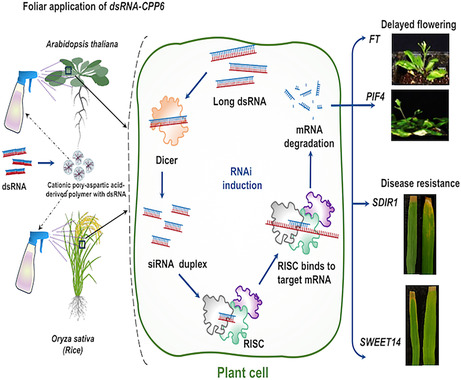

## 1 INTRODUCTION

Many abiotic and biotic factors such as drought, temperature, insects, bacteria, fungi and viruses limit plant growth, development and yield (Kamthan et al., 2015). Several omics and functional studies have been performed to identify the factors that limit plant adaptation to stress and productivity (Mittler & Blumwald, 2010; Tester & Langridge, 2010). Many small molecules, plant growth regulators and pesticides to counter these limitations have been identified, although their functions may be reversible and they may require repeated applications to achieve the desired efficacy (Margaritopoulos et al., 2020; Shah & Shad, 2020). Genetic manipulation of plant traits adversely affected by abiotic and biotic stresses have emerged as potential targets for crop improvement. As there are strong biosafety regulations and limited public acceptance of genetically modified (GM) crops, the development of transgene‐free plants is desirable but is also laborious and time‐consuming (Lusser et al., 2012). In this context, instantaneous and easy‐to‐execute strategies are needed. RNAi technology has been used for functional studies of genes and double‐stranded RNA (dsRNA) application has emerged as a highly promising technique to target genes to manipulate plant processes and improve crop protection against insects, fungi and viruses (Ibrahim et al., 2011; Simón‐Mateo & García, 2011; Xin et al., 2010). DICER‐LIKE proteins process the dsRNA inside the cells to produce small interfering RNAs (siRNAs), which are recognized by Argonaute (AGO) proteins to form RNA‐induced silencing complexes (RISC) (Vergani‐Junior et al., 2021). RISC along with siRNAs base pair with complementary mRNA to cleave the transcript or inhibit its translation (Fire et al., 1998). RNAi‐based technologies are considered low risk and could reduce the usage of chemical pesticides to attain sustainability goals.

The topical application of dsRNA can control plant viruses such as pepper mottle virus, tobacco mosaic virus and bean common mosaic virus (Konakalla et al., 2016; Mitter, Worrall, Robinson, Xu, et al., 2017; Tenllado et al., 2003; Worrall et al., 2019). *MoDES1*, a host‐defence suppressor pathogenicity gene of *Magnaporthe oryzae* that has been targeted by application of exogenous dsRNA, confers resistance against fungal blast disease in rice (Sarkar & Roy‐Barman, 2021). Host‐induced gene silencing (HIGS) strategies have been effectively used against pathogens and pests such as viruses, insects, fungi and nematodes (Koch & Wassenegger, 2021). The success of HIGS depends on the efficiency of plant DCL proteins involved in processing siRNAs, the uptake ability of siRNAs by the pathogen and feeding behaviour of animal vectors (Wang et al., 2015). HIGS vectors with hairpin sequences have been used to express dsRNAs in plants. dsRNA targeting the *CarE* gene of *Sitobion avenae* expressed in wheat delayed larval growth (Xu et al., 2014). Transgenic plants expressing dsRNA (*CYP3‐RNA*) targeting all three copies of the *Fusarium graminearum CYP51* gene (*FgCYP51A, FgCYP51B, FgCYP51C*) in *Arabidopsis* and barley (*Hordeum vulgare*) inhibited fungal infection through HIGS (Koch et al., 2019; Nowara et al., 2010). The success of HIGS leads to the direct delivery of siRNAs against pathogen/insect genes using environment‐friendly spray‐induced gene silencing (SIGS) approaches. The exogenous application of dsRNAs targeting *S. avenae structural sheath protein* (*Shp*), involved in aphid feeding behaviour, protected barley plants (Biedenkopf et al., 2020). Targeting *M. oryzae MoDES1*, an innate defence suppressor of rice, using SIGS confers partial resistance to the fungus (Sarkar & Roy‐Barman, 2021). Although dsRNAs have been used to target virus, insect and fungal genes, attempts to target plant endogenous genes and bacterial genes are limited. RNAi technologies have successfully improved crop yield by targeting agronomically important traits like height, branching and increased biomass. Knockdown of *OsDWARF4* in rice caused improved yield as plants possess short height and erect leaf architecture with increased photosynthesis in lower leaves (Feldmann, 2006). Maize *Corngrass1* (*Cg1*) miRNA overexpression in *Arabidopsis* and switchgrass prolongs the vegetative phase, delays flowering, increases biomass with 250% more starch and improved digestibility (Chuck et al., 2011).

In rice, bacterial leaf blight (BLB) disease is caused by *Xanthomonas oryzae* pv. *oryzae* (Xoo) can severely affect yield. Streptomycin and copper oxychloride‐based pesticides have been used for crop protection; however, prolonged usage of pesticides leads to the development of resistant strains (Prasad et al., 2018). Therefore, there is a demand for alternative ways to control pathogenicity and to improve agronomic traits. Several genes may be targeted to manipulate plant growth and improve resistance against Xoo (Bakade et al., 2021; Pal et al., 2022; Vemanna et al., 2019). Overexpression of transcription factor *OsWRKY62* compromised the basal defence and *Xa21*‐mediated resistance to Xoo (Peng et al., 2008). OsWAK12 is a negative regulator for rice blast disease that altering basal resistance (Delteil et al., 2016). Xoo secretes transcription‐activator‐like effectors (TALEs) into plants that bind to the promoter of *SWEET14* (Bezrutczyk et al., 2018; Oliva et al., 2019; White et al., 2009). *Arabidopsis* plants overexpressing *salt‐ and drought‐induced RING box* (*SDIR1*) are susceptible to *Pseudomonas syringae* pv. *tomato* (Pst) and *sdir1* mutants are resistant (Ramu, Oh, et al., 2021; Ramu, Pal, et al., 2021). Targeting such negative regulators using dsRNA is likely to provide an opportunity to manipulate plant growth, immunity and productivity. However, the major challenge that remains is the stability and regulated delivery of the dsRNA to the site of action, because naked dsRNA is highly prone to degradation in the natural environment due to conditions like sunlight, UV light and pH (Christiaens et al., 2020). Therefore, attempts have been made to deliver dsRNA using nanoparticles. Delivery of dsRNA with clay nanosheets provides sustained protection against cucumber mosaic or pepper mild mottle viruses in local and systemic tissues (Mitter, Worrall, Robinson, Li, et al., 2017). The foliar application of dsRNA conjugated with bioclay effectively protected plants against the phloem‐feeding pest *Bemisia tabaci* (Mitter, Worrall, Robinson, Li, et al., 2017). The dsRNA is processed into 21–22 nucleotide (nt) siRNAs by whitefly RNAi machinery, which leads to homologous transcript degradation and thus insect mortality (Jain et al., 2022).

One challenge in HIGS is the systemic spread of the activated RNAi machinery. To achieve the maximum effectiveness of gene silencing, robust nanopolymers with systemic movement ability are required to deliver dsRNA. We report that cationic poly‐aspartic‐derived polymer (CPP) could effectively encapsulate dsRNA at lower concentrations than other reported polymers (Das & Sherif, 2020). CPP6 facilitated systemic spread of dsRNAs in plants following infiltration, foliar spray and root uptake. *Arabidopsis* plants overexpressing *GFP* were targeted by dsRNA and showed prolonged suppression of GFP fluorescence. The flowering‐associated genes *flowering locus T* (*FT*) and *phytochrome interacting factor 4* (*PIF4*) in *Arabidopsis* were targeted by SIGS. *Phytoene desaturase* (*PDS*) and *bZIP23* transcription factor were targeted through root uptake in rice. Foliar SIGS targeted against *OsSWEET14* and *OsSDIR1* provided resistance against Xoo infection. CPP6 nanopolymers could effectively deliver dsRNAs into the plants and prolonged silencing of the target gene through RNAi machinery activation. A low amount of dsRNA could successfully provide durable resistance against Pst and Xoo.

## 2 RESULTS

### 2.1 Nanoformulation‐based topical delivery of dsRNA into plants

**FIGURE 1:**
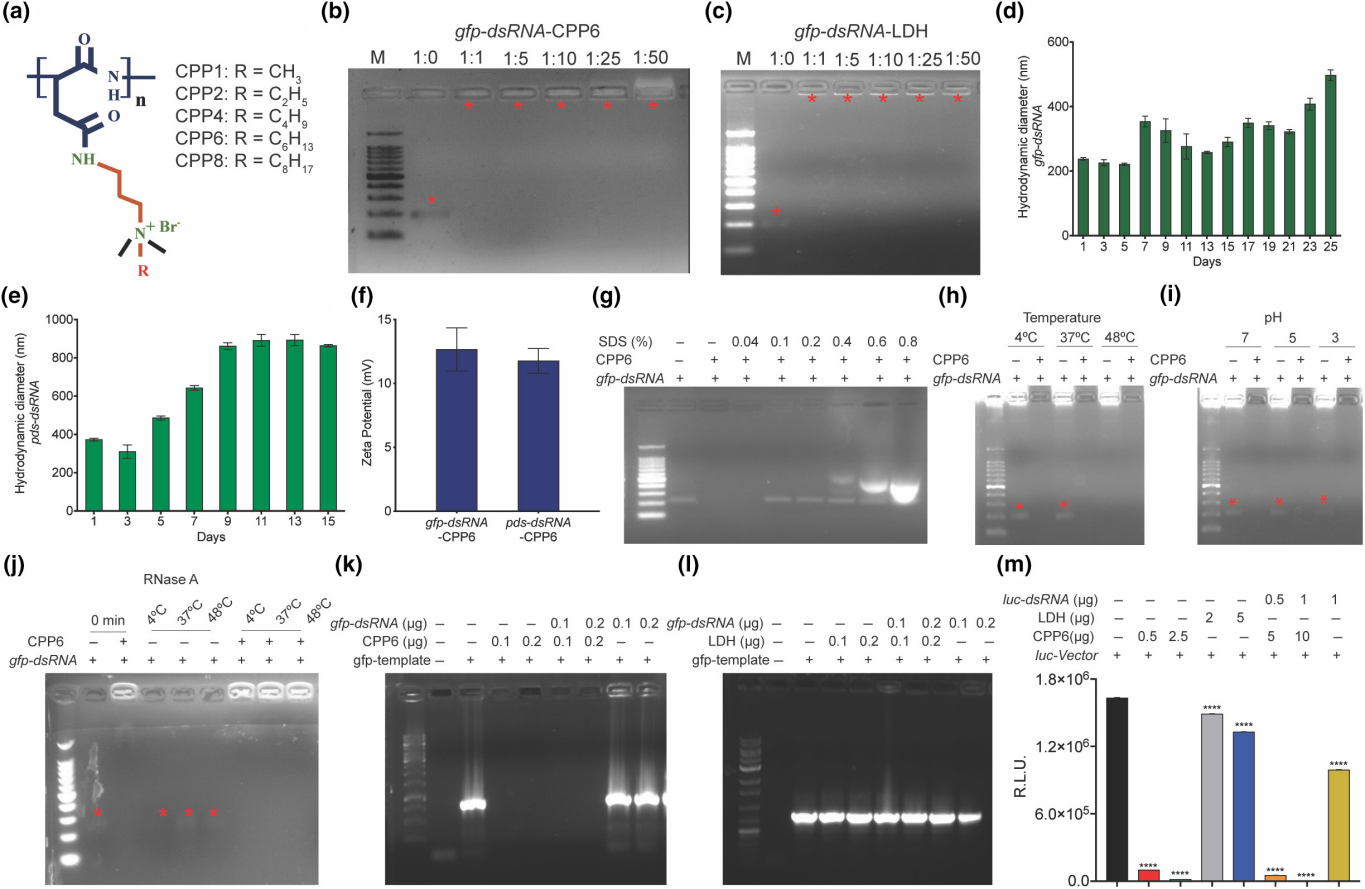
Cationic poly‐aspartic acid‐derived polymers (CPP) provide double‐stranded RNA (dsRNA) stability. (a) Structure of different CPPs. Polymer‐*gfp*‐dsRNA complex formation with (b) CPP6 and, (c) layered double hydroxide (LDH) nanoparticles. Five hundred nanograms of in vitro‐synthesized *GFP*‐dsRNA was incubated for 30 min at room temperature with different ratios of polymers and loaded on 2% agarose gel. Naked *gfp*‐dsRNA is visible in the gel whereas *gfp*‐dsRNA‐CPP6 and *gfp*‐dsRNA‐LDH complex remain in the wells. Hydrodynamic diameter size (nm) of (d) *gfp*‐dsRNA‐CPP6 and (e) *pds*‐dsRNA‐CPP6 complexes was measured after incubating dsRNA with polymer for 30 min at room temperature. (f) The zeta potential (ZP) of dsRNA‐CPP6 complexes of *GFP* and *PDS* fragments. (g) Release of *gfp*‐dsRNA using SDS from polymer complexes. The *gfp*‐dsRNA‐CPP6 complexes were treated with different concentrations of SDS for 30 min and resolved on 2% agarose gel. + indicates 5 μg of CPP6 and 500 ng of dsRNA in each reaction. The big bright bands are higher concentrations of SDS forming micelles with ethidium bromide, giving fluorescent anionic bands that migrate down the gel. Stability of dsRNA and *gfp*‐dsRNA‐CPP6 complexes, (h) at different temperatures, (i) different pH and, (j) with RNase A treatment. Efficiency in interfering with DNA accessibility for amplification by (k) CPP6 and (l) LDH nanoparticles. In a PCR, different combinations of *gfp*‐dsRNA were complexed with CPP6, LDH nanoparticle and added to the PCR with *gfp* template. (m) In vitro translation efficiency in the presence of CPP6, LDH nanoparticle with *luc*‐dsRNA. *luc*‐dsRNA and CPP6, complexes in the reaction mixture. Error bars indicate values of means ± *SE* from three biological replicates, experiments repeated two times with similar results. The significance of differences was examined using Student's *t* test (*⍺* = 0.05, ****p < 0.0001).

Naked dsRNA delivery into plants is challenging in natural environmental conditions as it is highly prone to degradation by UV light, microorganisms and washout in rain. A range of polymeric nanocarriers having structural flexibility for modulation, bioconjugation, degradability and stability in varied biological systems were utilized to deliver dsRNA. Five different cationic poly‐aspartic acid‐derived polymers (CPP1, CPP2, CPP4, CPP6, and CPP8), biocompatible and biodegradable with varied hydrophobicity, were synthesized previously (Figure 1a) and can effectively deliver nucleic acids into mammalian cells (Yavvari et al., 2019). No inhibitory effect on shoot and root length by these different CPPs on rice seedlings at 5, 25, 50 and 75 μg/mL concentrations were observed (Figure S1). The CPPs did not interfere with the growth and development of the seedlings. The CPP6‐based formulation was previously reported to be the most efficient for intracellular delivery of siRNAs against the SUMOylation machinery of the mammalian mouse model to reduce gut inflammation. CPP6 can form polyplexes with siRNA and maintained moderate positive zeta potential (ZP) with the highest transfection efficiency in the mammalian system (Yavvari et al., 2019). Thus, we chose CPP6 to deliver dsRNAs into plant cells. dsRNA targeting *GFP* was used to test the mass ratio required to stabilize nucleic acids in the complex. CPP6 conjugated with 500 ng of dsRNA was incubated in a 1:1, 1:5, 1:10, 1:25 and 1:50 wt/wt ratio at room temperature for 30 min and resolved on an agarose gel. Even at 1:1 ratio, the polymer could encapsulate dsRNA quite effectively (Figure 1b). The layered double hydroxide (LDH) polymer efficiency was also assessed at 1:1, 1:5, 1:10, 1:25 and 1:50 wt/wt ratio and showed similar encapsulation of dsRNA (Figure 1c). The transfection efficiency of polymers in delivering siRNAs in the mammalian systems is effective with a 1:10 ratio (Yavvari et al., 2019). At lower ratios, the dsRNA may be released quickly and not efficiently silence the plant's endogenous genes. Therefore, a 1:10 ratio of dsRNA:CPP6 was chosen for dsRNA delivery and durable effectiveness in plants to silence the target genes. A hydrodynamic diameter (D_H_) size range of 200–500 nm was measured for CPP6 with *gfp*‐dsRNA and *pds*‐dsRNA (Figure 1d,e). Similarly, the ZP of CPP6 with dsRNA of *gfp* and *pds* was >10 mV (Figure 1f). To assess the release of dsRNA from CPP complexes, different concentrations of sodium dodecyl sulphate (SDS) were used; 0.1% SDS could completely release the dsRNA (Figure 1g). Higher concentrations of SDS formed micelles and interacted with ethidium bromide, giving fluorescent anionic bands that migrated down the agarose gel, as reported by Holt et al. (2020). The success of exogenous application of dsRNA on plants depends on systemic translocation and prolonged availability of dsRNA to activate RNAi machinery. The stability of dsRNA‐CPP6 complex and naked dsRNA was assessed by incubation at 4, 37 and 48°C for 10 days. Naked dsRNA at 48°C showed degradation after 4 days and was not detectable after 10 days, whereas the dsRNA‐CPP6 complex showed negligible release of dsRNA from the polymer (Figures 1h and S2a). Similarly, the stability of the dsRNA‐CPP6 complex at pH 7, 5 and 3 was assessed. Naked dsRNA was degraded at pH 3 after 10 days whereas the dsRNA‐CPP6 complex showed no release of dsRNA, suggesting the efficiency of CPP6 in nucleic acid protection (Figures 1i and S2b). The stability of dsRNA‐CPP6 complex and naked dsRNA was also assessed by incubating with RNase A at 4, 37 and 48°C (Figures 1j and S2c,d). RNase A treatment did not influence either the release or degradation of dsRNA.

CPP6 and LDH polymer complexes provide stability to dsRNA, and so we investigated whether these polymers could interfere with DNA template accessibility for polymerase activity in replication, transcription and translation, which is also useful for gene silencing. PCRs containing the *GFP* template, CPP6 and *gfp*‐dsRNA‐CPP6 complexes produced no amplified fragments. In contrast, naked *gfp*‐dsRNA did not inhibit the reaction, suggesting that CPP6 interfered with template DNA accessibility for the amplification (Figures 1k and S2d). However, with LDH polymer at 100 ng and 200 ng, PCR amplification of the genes was unaffected, suggesting their inefficiency in interfering with *GFP* template accessibility (Figure 1l). To assess whether CPP6 and LDH polymers interfere with *luciferase* (*luc*)‐dsRNA and inhibit the translation process, an coupled transcription and translation assay was performed. CPP6, at 500 ng in a coupled transcription/translation reaction in vitro, reduced the relative luminescence activity of luciferase, whereas LDH polymer, even at 2000 ng, showed higher luminescence compared to the control reaction. The *luc*‐dsRNA‐CPP6 complex showed a significant reduction in relative luminescence compared to the control, whereas naked dsRNA showed some extent of reduction (Figure 1m). This suggests that CPP6 can interfere in the suppression of genes during the translation process and demonstrates that dsRNA‐CPP6 affects gene silencing at multiple levels.

**FIGURE 2:**
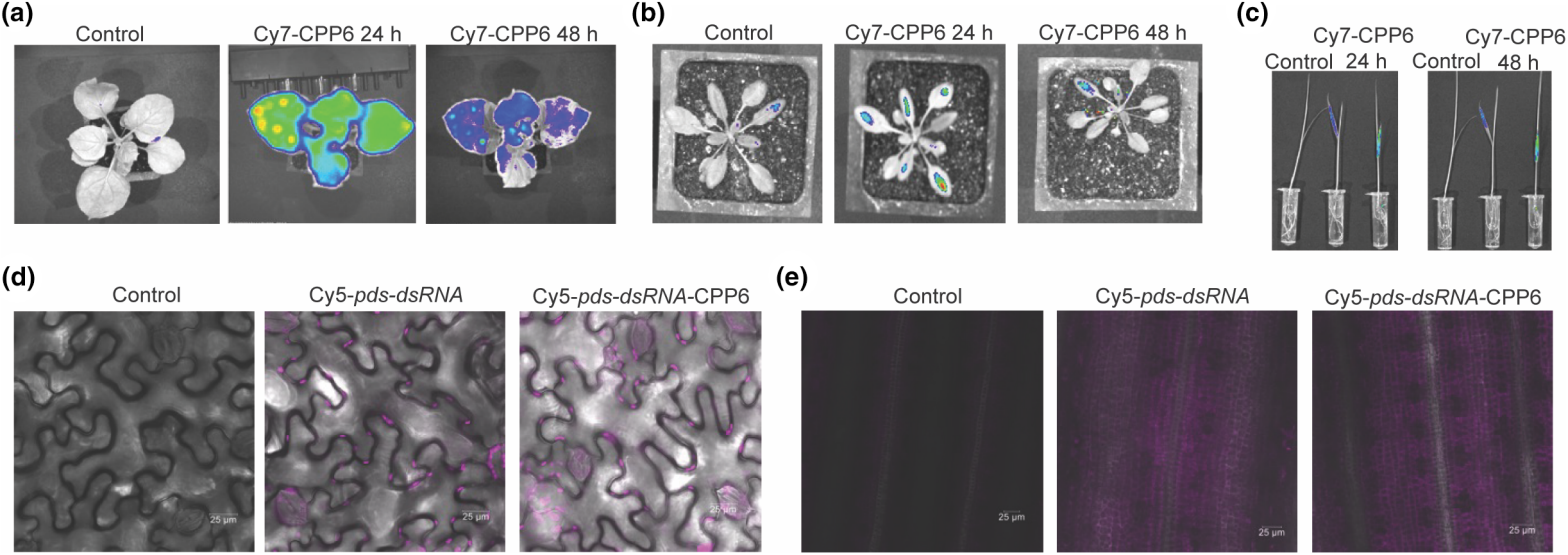
CPP6 facilitates double‐stranded RNA (dsRNA) uptake in plants. In planta uptake and translocation of CPP6 polymer. The Cy7‐fluorophore was attached to CPP6 and infiltrated into (a) *Nicotiana benthamiana* and (b) *Arabidopsis* then visualized using a near‐infra‐red (NIR) imaging system at 24 and 48 h post‐infiltration (hpi). (c) Uptake of CPP6‐Cy7 through roots by rice seedlings. NIR signals were visualized at 24 and 48 hpi. Visualization of *pds*‐dsRNA in plant leaves by confocal microscopy. *pds*‐dsRNA labelled with Cy5‐dUTP, Cy5‐dUTP‐dsRNA‐CPP6 complexes, (d) in *N. benthamiana* at 24 hpi and (e) uptake through roots in rice at 24 hpi.

To test CPP6 efficiency in plant cellular uptake and systemic spread to different plant parts, Cy7‐labelled CPP6 was infiltrated into *Nicotiana benthamiana* and *Arabidopsis thaliana* leaves. Fluorescence was detected in leaves after 24 and 48 h (Figure 2a,b). Near‐infra‐red (NIR) signals were visible in distal leaves, indicating the systemic movement of the polymer in plants. The uptake of the polymer through roots was assessed in rice seedlings; NIR signals were visible in the top leaves after 24 and 48 h (Figure 2c). To visualize dsRNA uptake in plants, Cy5‐labelled naked *pds*‐dsRNA and Cy5‐*pds*‐dsRNA‐CPP6 was infiltrated in *N. benthamiana* leaves; signals were detected 24 h post‐infiltration (hpi) in plant cells (Figure 2d). Similarly, rice seedlings were immersed in Cy5‐dsRNA and Cy5‐dsRNA‐CPP6; fluorescence in the rice sheath at 24 hpi indicated uptake of labelled dsRNA through the roots (Figure 2e).

### 2.2 Silencing of *GFP* in *Arabidopsis* using dsRNA‐CPP6

**FIGURE 3:**
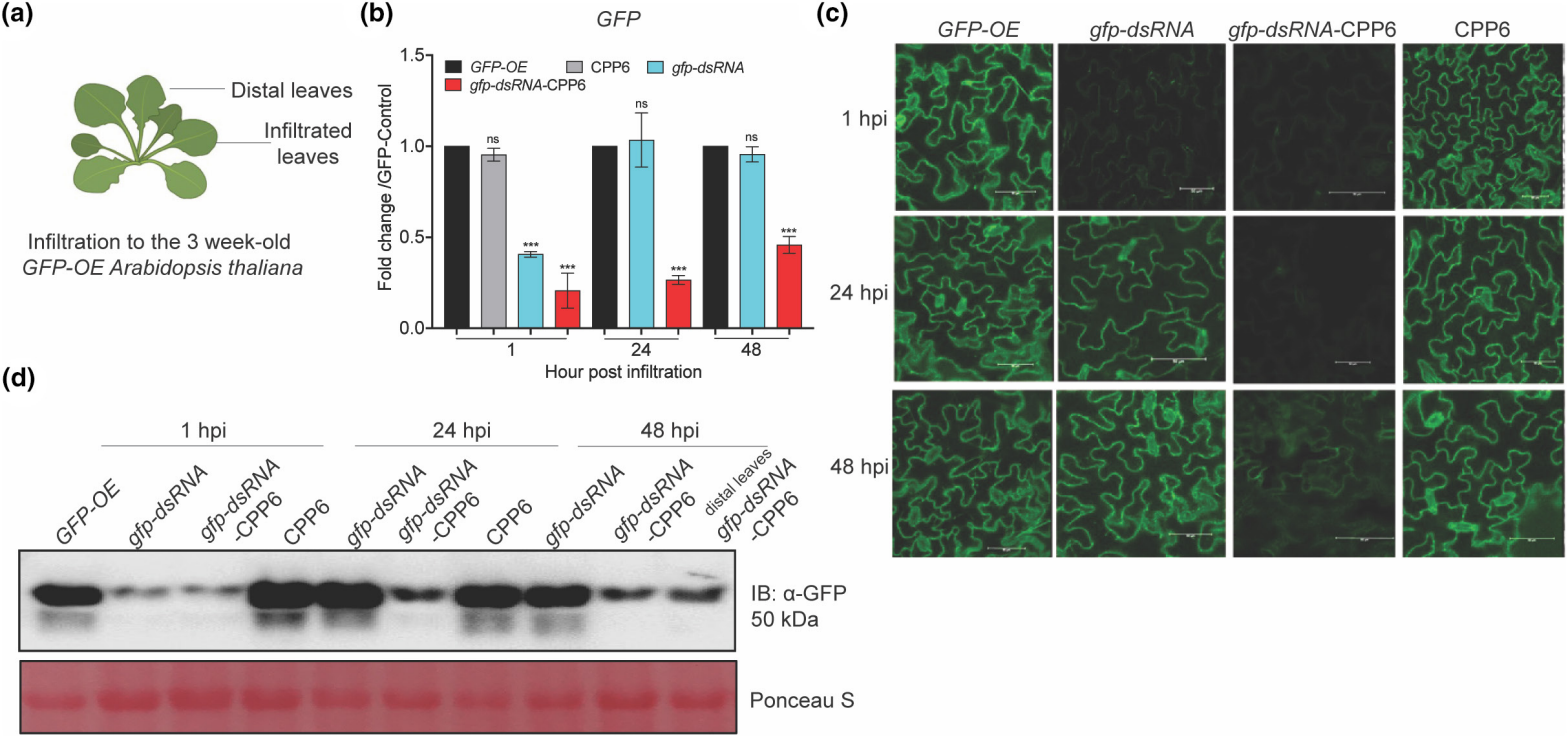
Silencing of green fluorescent protein (*GFP*) transgene in *Arabidopsis* using *gfp*‐dsRNA. (a) Schematic representation of the application of *gfp*‐dsRNA‐CPP6 through syringe infiltration in transgenic *RPL10‐GFP‐OE Arabidopsis* plants. Three‐week‐old plants were infiltrated with *gfp*‐dsRNA or *gfp*‐dsRNA‐CPP6, control plants were mock‐treated with water. (b) *GFP* transcript levels in *gfp*‐dsRNA‐ and *gfp*‐dsRNA‐CPP6‐treated plants at 1, 24 and 48 h post‐infiltration (hpi). (c) Confocal microscopic images showing GFP fluorescence signals in *gfp*‐dsRNA‐and *gfp*‐dsRNA‐CPP6‐infiltrated leaves at 1, 24 and 48 hpi. (d) Immunoblot (IB) showing RPL10‐GFP levels in leaves at 1, 24 and 48 hpi from the infiltration site and in distal leaves. The total protein was isolated from the leaves, and equal concentrations were loaded for each sample on SDS‐PAGE. Anti‐GFP antibody was used to detect the levels of protein. Ponceau S staining was done to analyse the protein normalization. Error bars indicate values of means ± *SE* from three biological replicates. The significance of differences was examined using Student's *t* test (*⍺* = 0.05, ***p < 0.001).

To test the efficiency of dsRNA in silencing plant endogenous genes, transgenic *Arabidopsis* plants overexpressing the *green fluorescent protein* gene (*GFP‐OE*) were developed. Naked *gfp*‐dsRNA and *gfp*‐dsRNA‐CPP6 complex were syringe‐infiltrated into 3‐week‐old *GFP‐OE* plants (Figure 3a). *GFP* transcript levels were reduced in naked *gfp*‐dsRNA‐ and *gfp*‐dsRNA‐CPP6‐treated plants compared to untreated plants. At 1 h post‐infiltration (hpi), naked *gfp*‐dsRNA‐treated plants showed >2‐fold reduction, whereas *gfp*‐dsRNA‐CPP6‐treated plants showed >4‐fold reduction of *GFP* transcripts. After 24 hpi, *GFP* transcript levels in naked *gfp*‐dsRNA‐treated plants were comparable to control plants, whereas *gfp*‐dsRNA‐CPP6‐infiltrated plants showed >4‐fold reduction in *GFP* transcripts. At 48 hpi, *GFP* transcript levels in *gfp*‐dsRNA‐CPP6‐infiltrated plants were reduced >2‐fold compared to control plants (Figure 3b). GFP fluorescence in CPP6‐only infiltrated plants was equivalent to the *GFP‐OE* control plants, suggesting no interference by the CPP6 in gene silencing. In the naked *gfp*‐dsRNA‐infiltrated plants, signals were reduced at 1 hpi; however, by 24 hpi the GFP signals had recovered. The *gfp*‐dsRNA‐CPP6‐infiltrated plants showed significantly reduced GFP fluorescence even at 48 hpi (Figure 3c). The effect of dsRNA was assessed at the protein level using GFP‐specific antibodies by western blotting at different time points. At 1 hpi, in *gfp*‐dsRNA‐ and *gfp*‐dsRNA‐CPP6‐infiltrated plants, GFP levels were reduced drastically compared to the *GFP‐OE* control plants. At 24 hpi, GFP levels recovered to normal in naked dsRNA‐infiltrated plants, whereas *gfp*‐dsRNA‐CPP6‐infiltrated plants showed reduced GFP levels even at 48 hpi, suggesting CPP6 could provide prolonged effectiveness for gene silencing. The GFP in distal leaves of *gfp*‐dsRNA‐CPP6‐infiltrated plants showed a reduction of protein levels compared to the *GFP‐OE* control plants, confirming the systemic movement and extended availability of dsRNA due to CPP6 (Figure 3d).

### 2.3 Foliar application of dsRNA‐CPP6 can regulate flowering genes in *Arabidopsis*

**FIGURE 4:**
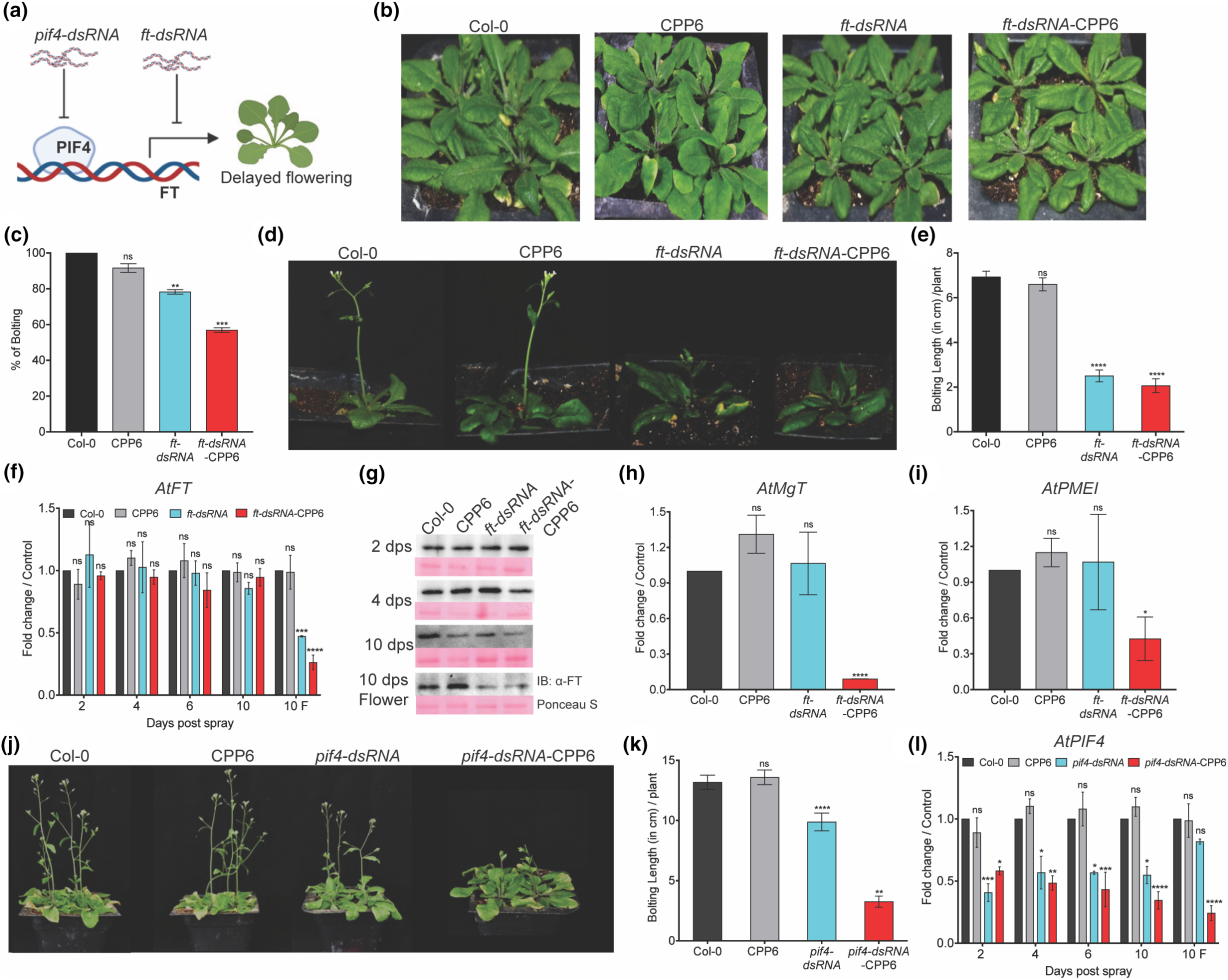
Delayed flowering in *Arabidopsis* Col‐0 plants targeting *flowering locus T* (*FT*) and *phytochrome interacting factor* 4 (*PIF4*) through foliar application of double‐stranded RNA (dsRNA). (a) Schematic representation of *FT* and *PIF4* role in flowering regulation and application of specific dsRNA targeting these genes to delay the flowering. dsRNA concentration of 125 ng/plant with CPP6 was sprayed before bolting and phenotypic characters were observed. Plants sprayed with water or CPP6 were used as the controls. A minimum of 20 plants for each treatment was used. (b) Phenotype of delayed bolting initiation in plants sprayed with *ft*‐dsRNA and *ft*‐dsRNA‐CPP6 complex at 48 h post‐spraying (hps). (c) Percentage of plants showing bolting initiation in *ft*‐dsRNA‐ and *ft*‐dsRNA‐CPP6‐sprayed plants at 48 hps. Error bars indicate the average percentage from three experiments with a minimum of 20 plants. (d) Phenotype of plants sprayed with *ft*‐dsRNA or *ft*‐dsRNA‐CPP6 compared to water‐ and CPP6‐sprayed plants after 10 days post‐spraying (dps). (e) Bolting length in *ft*‐dsRNA‐ and *ft*‐dsRNA‐CPP6‐sprayed plants at 10 dps. (f) Expression of *FT* in leaf samples collected after 2, 4, 6, 10 dps and floral parts at 10 dps compared to water‐ and CPP6‐sprayed plants. (g) FT levels at different time intervals were assessed by immunoblotting using FT‐specific antibodies. (h) Expression of predicted off‐target genes *Magnesium transporter* (*MgT*) and (i) *Pectin methyl transferase inhibitor* (*PMEI*) at 10 dps in floral parts, (j) phenotype of *PIF4* targeted plants sprayed with water, CPP6, *pif4*‐dsRNA or *pif4*‐dsRNA‐CPP6 at 7 dps. (k) Bolting length at 10 dps in plants sprayed with *pif4*‐dsRNA, *pif4*‐dsRNA‐CPP6 or water. (l) Expression of *PIF4* in leaf samples collected at 2, 4, 6, 10 dps and floral parts at 10 dps compared to water‐ and CPP6‐sprayed plants. Error bars indicate values of means ± *SE* from three biological replicates. A minimum of 24 plants was used with similar results repeated three times. The significance of differences were examined using Student's *t* test (*⍺* = 0.05, *p < 0.05, **p < 0.01, ***p < 0.001, ****p < 0.0001).

The major focus of our study was to target the plant endogenous genes to modulate plant processes related to growth, development, stress and disease resistance in a transient manner. *FT* and *PIF4* in *Arabidopsis* were targeted by respective dsRNA‐CPP6, which caused a delay in flowering time and enhanced biomass. FT is known to translocate through the phloem from leaves to the shoot apical meristem, causing a switch from vegetative to reproductive organ development. *FT* overexpression in various species accelerates flowering. PIF4, a bHLH transcription factor, regulates *FT* activation through direct binding to its promoter, and RNAi lines have delayed flowering (Kumar et al., 2012). To delay flowering and improve biomass in *Arabidopsis*, *FT* and *PIF4* transcripts were targeted through dsRNA (Figure 4a). *Arabidopsis* Col‐0 plants, before bolting, were exogenously sprayed with 125 ng/plant *ft*‐dsRNA and *ft*‐dsRNA‐CPP6. At 48 h post‐spraying (hps), fewer plants sprayed with naked *ft*‐dsRNA or *ft*‐dsRNA‐CPP6 showed early bolting initiation compared to water‐ and CPP6‐sprayed plants (Figure 4b,c). At 10 dps, bolting length was reduced >2‐fold and >4‐fold in *ft*‐dsRNA‐ and *ft*‐dsRNA‐CPP6‐treated plants, respectively (Figure 4d,e). At 2, 4, 6 and 10 dps, *FT* transcript levels were unchanged in leaves, whereas in floral parts at 10 dps transcript levels were reduced >2‐fold and >4‐fold in plants sprayed with *ft*‐dsRNA and *ft*‐dsRNA‐CPP6, respectively (Figure 4f). The FT protein levels were assessed in treated plants at different time points and reduction was observed at 4 dps and 10 dps in floral parts of *ft*‐dsRNA‐CPP6‐sprayed plants compared to water‐ and CPP6‐sprayed plants. In the naked *ft*‐dsRNA‐treated plants, the reduction was observed only in floral parts at 10 dps (Figure 4g). The predicted off‐target gene *Magnesium transporter* (*MgT*) levels were reduced in *ft*‐dsRNA‐treated plants (Figure 4h), which may reflect a role in floral bud development and fertility (Pabón‐Mora et al., 2022). Delayed flowering of the dsRNA‐treated plants does not signal the induction of *MgT* and thus less transcript levels were observed (Figure S3). Expression of another predicted off‐target gene *Pectin methyl esterase inhibitor* (*PMEI*) was also reduced in *ft*‐dsRNA‐CPP6‐treated plants (Figure 4i). The higher expression of *PMEI* in *Arabidopsis* inhibits floral organ primordia (Peaucelle et al., 2008).

The upstream regulator of *FT*, PIF4, was targeted by *pif4*‐dsRNA. Three‐week‐old *Arabidopsis* plants were sprayed with 125 ng/plant *pif4*‐dsRNA; at 7 dps bolting length was reduced by >4‐fold in *pif4*‐dsRNA‐CPP6‐ and >1‐fold in *pif4*‐dsRNA‐treated plants compared to control and CPP6‐alone sprayed plants (Figure 4j,k). The transcript levels of *PIF4* were reduced >2‐fold from 2 dps to 10 dps in *pif4*‐dsRNA, whereas in *pif4*‐dsRNA‐CPP6‐sprayed plants up to 6 dps, >2‐fold reduction and at 10 dps >3‐fold reduction was observed in leaves as well as in floral organs (Figure 4l).

### 2.4 dsRNA uptake in rice seedlings can effectively silence genes

**FIGURE 5:**
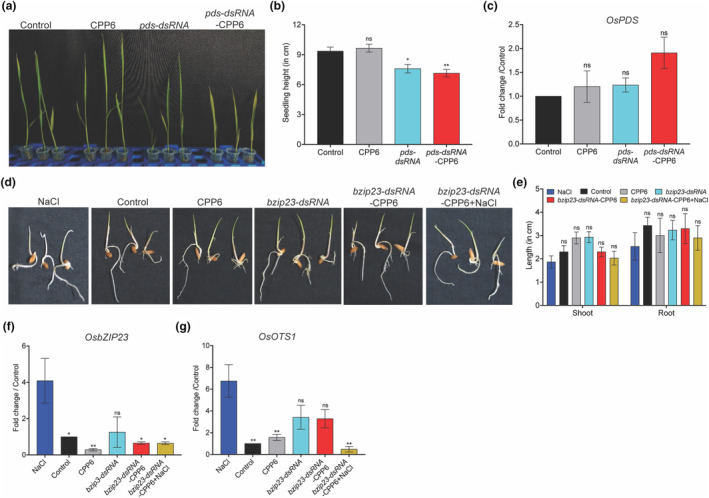
Effectiveness of dsRNA‐CPP6 targeting *phytoene desaturase synthase* (*PDS*) and *OsbZIP23* through root uptake in rice. (a) The phenotype of rice seedlings showing stunted, yellowing and albino leaves in *pds*‐dsRNA and *pds*‐dsRNA‐CPP6 treatments. Rice seedlings grown for 3 days in the light were transferred to microcentrifuge tubes in water. Two micrograms of *pds*‐dsRNA and *pds*‐dsRNA‐CPP6 was added to individual vials and kept in the dark for 10 days. Plants treated with water or CPP6 were used as controls. (b) Seedling height in *pds*‐dsRNA‐ and *pds*‐dsRNA‐CPP6‐treated plants compared to treatment with water and CPP6 10 days after treatment. (c) *PDS* transcript levels 48 h after treatment. Total RNA from leaf samples was isolated and converted to cDNA and used as template. (d) Phenotypic images of rice seedlings exposed to 150 mM NaCl stress, *bzip23*‐dsRNA, *bzip23*‐dsRNA‐CPP6 and *bzip23*‐dsRNA‐CPP6 + NaCl. Photographs were taken after 48 h of exposure. (e) Shoot and root length of treated seedlings were recorded 48 h after treatment. (f) Expression of *OsbZIP23* and (g) *bZIP23* transcription factor target gene *OTS1* in NaCl‐ and *bzip23*‐dsRNA‐treated samples. The pregerminated seedlings were treated with 150 mM NaCl for 24 h and then treated with 150 ng *bzip23*‐dsRNA, *bzip23*‐dsRNA‐CPP6 and allowed to grow for 48 h. A minimum of 10 seedlings was used for each treatment. Error bars indicate values of means ± *SE* from three biological replicates. The significance of differences was examined by Student's *t* test (*⍺* = 0.05, *p < 0.05, **p < 0.01).

To assess the effectiveness of dsRNA‐mediated silencing in rice, we targeted the *phytoene desaturase* (*PDS*) gene using seedling dip‐inoculation. Three‐day‐old seedlings were treated with 2 μg of naked *pds*‐dsRNA or *pds*‐dsRNA‐CPP6 and allowed to take it up through the roots. At 10 days post‐treatment (dpt), *pds*‐dsRNA‐treated seedlings showed reduced growth with a yellowish to albino phenotype (Figure 5a). The seedling height was reduced >1‐fold in *pds*‐dsRNA‐CPP6‐treated samples compared to CPP6‐alone treated and control seedlings (Figure 5b). *PDS* transcript levels did not show any reduction compared to control plants. However, a typical albino phenotype was observed (Figure 5c). *OsPDS* is required at higher levels during early‐stage development, thus transcript levels were not reduced; however, *pds*‐dsRNA‐CPP6‐sprayed plants showed an albino phenotype and reduced growth. We hypothesize that stress‐induced transcripts of negative regulators may be efficiently targeted through dsRNA. *OsbZIP23* transcription factor is upregulated in salt, drought and oxidative stress conditions (Pa et al., 2022; Sujitha et al., 2023). *OsbZIP23* was therefore targeted by dsRNA. The germinated rice seedlings were exposed to NaCl‐induced stress and treated with *bzip23*‐dsRNA‐CPP6. At 48 hpt, the growth of seedlings in NaCl was reduced; however, the growth of plants treated with naked *bzip23*‐dsRNA was not affected (Figure 5d,e). The expression of *bZIP23* under NaCl stress was triggered significantly compared to the control, and in polymer‐alone or *bzip23*‐dsRNA‐alone treated seedlings, no induction was observed. However, in NaCl‐exposed seedlings treated with *bzip23*‐dsRNA‐CPP6, the expression of *bZIP23* was significantly reduced, suggesting the effective silencing of the target gene (Figure 5f). The reduction in transcript levels also affected the expression of the *bZIP23* target gene, *Overlay Tolerant to Salt 1* (*OTS1*), which positively correlated under NaCl treatment (Figure 5g).

### 2.5 Improving plant protection by dsRNAs targeting disease susceptibility genes

**FIGURE 6:**
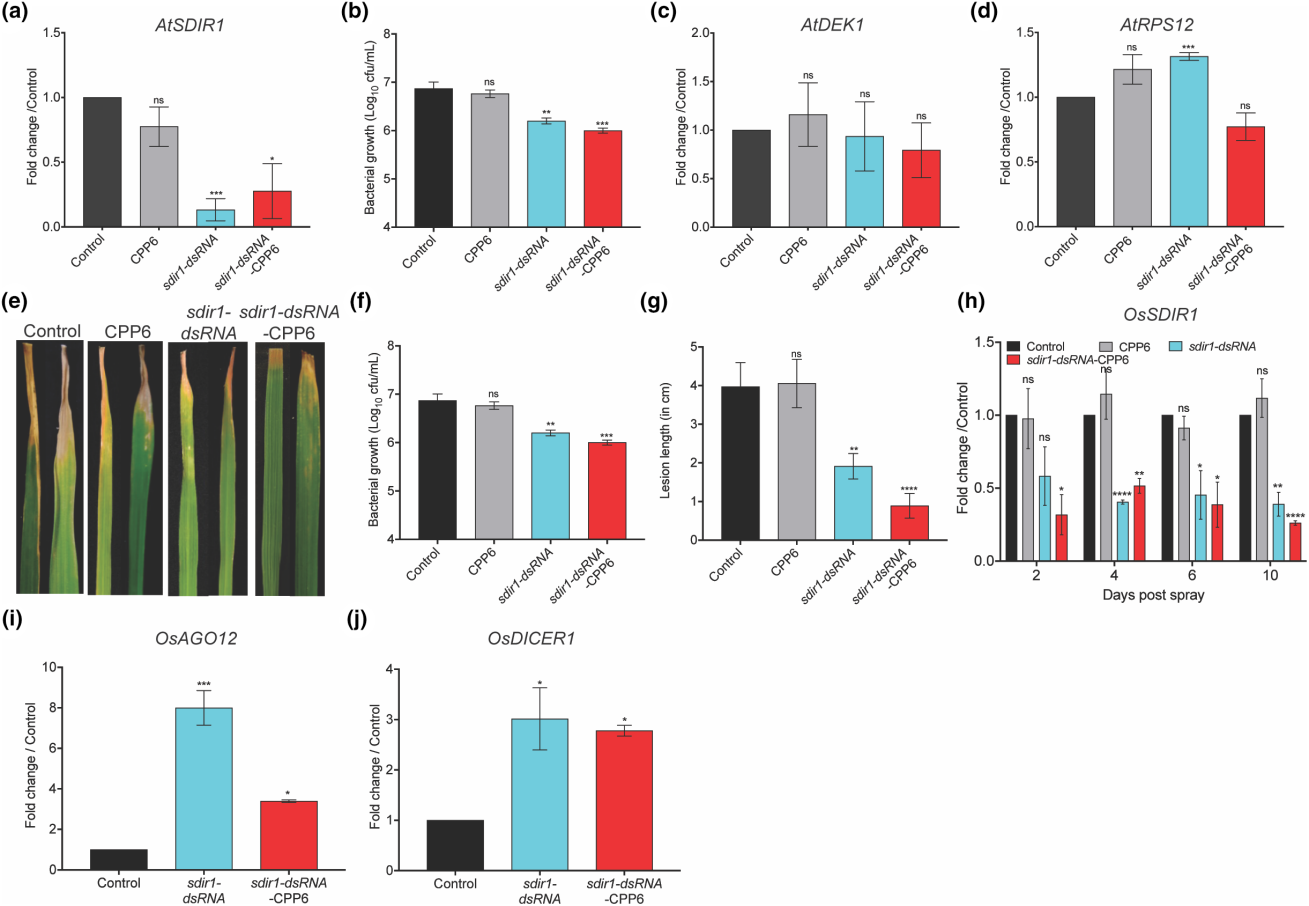
Plant disease susceptible genes were silenced using dsRNA‐CPP6 targeting *Salt and drought‐induced ring finger 1* (*SDIR1*) in *Arabidopsis* and rice to improve disease resistance. Three‐week‐old *Arabidopsis* plants were infiltrated with 10^6^ cfu/mL of *Pseudomonas syringae* pv. *tomato* mixed with CPP6, dsRNA or dsRNA‐CPP6. (a) Transcript levels of *SDIR1* in *sdir1*‐dsRNA‐ and *sdir1*‐dsRNA‐CPP6‐sprayed plants compared to water‐ and CPP6‐treated plants, (b) bacterial multiplication rate at 3 days post‐inoculation (dpi). (c) Expression of predicted off‐target *DEK1* and (d) *RPS12* at 3 dpi. TN1 rice plants (45 days old) were infected with 10^6^ cfu/mL *Xanthomonas oryzae* pv. *oryzae* using the leaf‐clipping method. After 24 h post‐inoculation (hpi) CPP6, *sdir1*‐dsRNA and *sdir1*‐dsRNA‐CPP6 were sprayed on plants. (e) Bacterial leaf blight disease symptoms at 10 dps, (f) bacterial multiplication rate at 48 hps and (g) lesion length in plants sprayed with *sdir1*‐dsRNA or *sdir1*‐dsRNA‐CPP6 compared to water and CPP6 treatments. (h) *SDIR1* expression in *sdir1*‐dsRNA‐ and *sdir1*‐dsRNA‐CPP6‐sprayed plants compared to water‐ and CPP6‐sprayed plants after 2, 4, 6 and 10 dps. (i) Expression of RNAi machinery‐associated genes *Argonaute* and (j) *Dicer* at 2 dps, in *sdir1*‐dsRNA‐ and *sdir1*‐dsRNA‐CPP6‐sprayed plants compared to water‐sprayed plants. Error bars indicate values of means ± *SE* from three biological replicates. The significance of differences was examined using Student's *t* test (*⍺* = 0.05, *p < 0.05, **p < 0.01, ***p < 0.001, ****p < 0.0001).

Mutation of *Salt and Drought‐Induced Ring Finger 1* (*SDIR1*) in *Arabidopsis* provides resistance against Pst (Ramu, Oh, et al., 2021). To assess the effectiveness of dsRNA‐CPP6 on *Arabidopsis* infected with Pst, bacterial culture containing *sdir1*‐dsRNA or *sdir1*‐dsRNA‐CPP6 was syringe infiltrated. *Arabidopsis* plants treated with dsRNA targeted to *AtSDIR1* showed reduced transcript levels, bacterial growth (Figure 6a,b) and a healthy phenotype (Figure S4a). The predicted off‐target *DEK domain‐containing chromatin‐associated protein‐encoding* transcripts were unaltered in all plants (Figure 6c). Expression of another off‐target gene, *RPS12*, involved in the pathogen defence response (Pal et al., 2022), was not changed in dsRNA‐CPP6‐treated plants (Figure 6d). We anticipated that silencing of *SDIR1* in rice through dsRNA should also provide resistance against BLB caused by Xoo. TN1, a susceptible rice variety, was grown for 45 days and infected with Xoo using the leaf‐clipping method. At 24 hpi, *sdir1*‐dsRNA (250 ng/leaf) or *sdir1*‐dsRNA‐CPP6 were sprayed. At 10 dps, plants treated with *sdir1*‐dsRNA or *sdir1*‐dsRNA‐CPP6 showed reduced disease symptoms compared to unsprayed control plants (Figure 6e). At 48 hps, bacterial multiplication rates reduced >1‐fold in *sdir1*‐dsRNA‐ and *sdir1*‐dsRNA‐CPP6‐sprayed plants compared to control plants (Figure 6f). The lesion lengths were reduced >2‐fold in *sdir1*‐dsRNA‐sprayed plants and to >4‐fold in *sdir1*‐dsRNA‐CPP6‐sprayed plants (Figure 6g). The transcript levels at 2 dps were downregulated >2‐fold and >3‐fold in *sdir1*‐dsRNA‐ and *sdir1*‐dsRNA‐CPP6‐treated plants, respectively. At 4 and 6 dps, transcript levels were reduced >2‐fold in *sdir1*‐dsRNA‐ and *sdir1*‐dsRNA‐CPP6‐treated plants compared to control plants. At 10 dps *SDIR1* transcripts were reduced >2.5‐fold and >3‐fold in *sdir1*‐dsRNA‐CPP6‐treated plants (Figure 6h). This experiment was repeated multiple times with similar phenotypes; bacterial growth and lesion lengths were reduced. However, sometimes we did not see reduced transcription of *SDIR1* similar to *PDS* (Figure S4b). The activation of the silencing machinery was assessed by quantifying the expression of *Dicer‐like* (*DICER*) and *Argonaute* (*AGO12*) expression. Expression of *AGO12* and *DICER1* were upregulated >2‐fold in *sdir1*‐dsRNA‐ and *sdir1*‐dsRNA‐CPP6‐treated plants, suggesting that the RNAi machinery was activated in plants with a disease‐resistance phenotype (Figure 6i,j).

**FIGURE 7:**
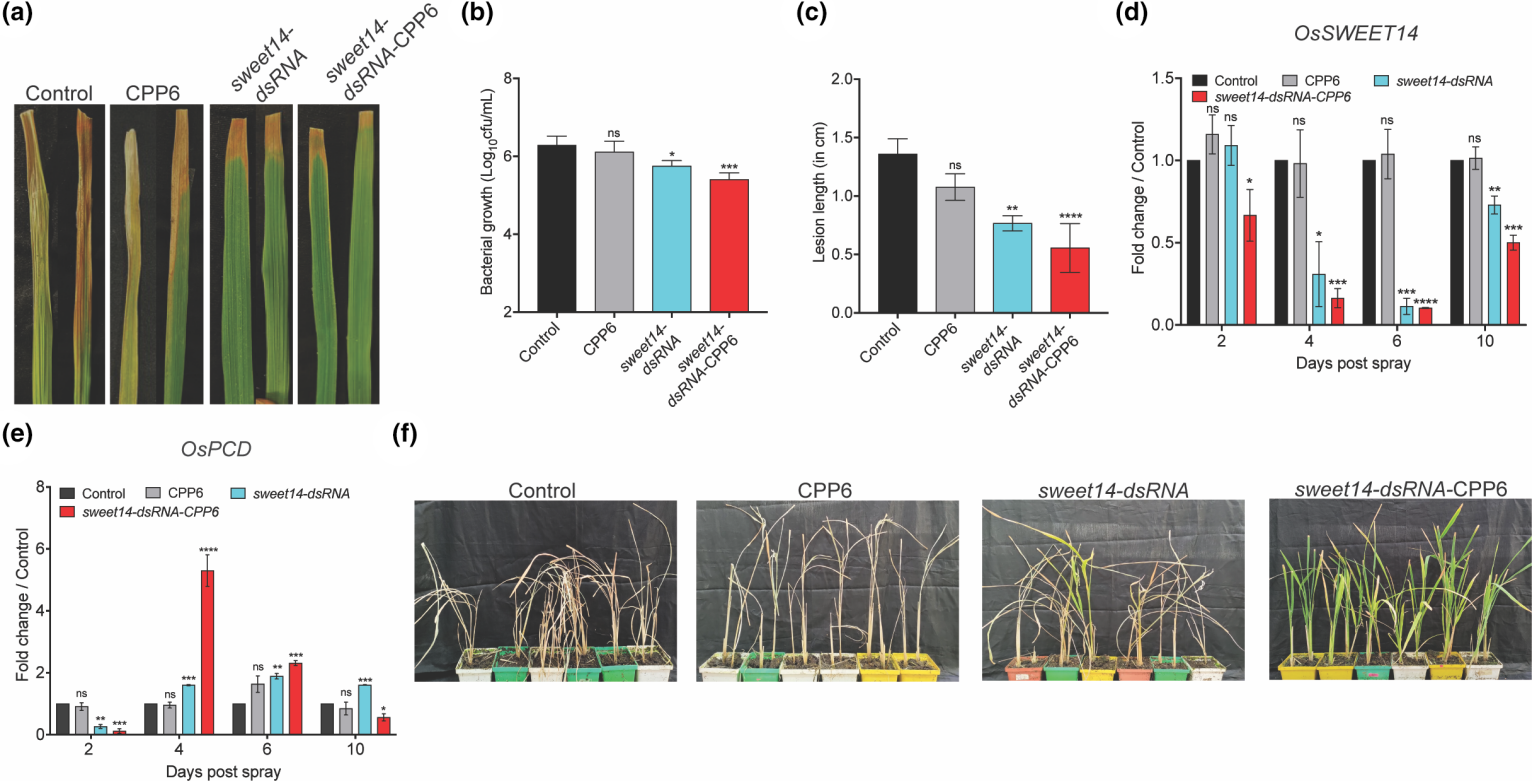
Double‐stranded RNA (dsRNA) targeting the *SWEET14* gene improved disease resistance against *Xanthomonas oryzae* pv. *oryzae* (Xoo). Rice TN1 plants (45 day old) were infected with 10^6^ cfu/mL Xoo using the leaf‐clipping method and 24 h post‐inoculation (hpi), CPP6, *sweet14*‐dsRNA or *sweet14*‐dsRNA‐CPP6 was sprayed on plants. (a) Bacterial blight disease symptoms at 10 days post‐spraying (dps) in leaves sprayed with *sweet14*‐dsRNA or *sweet14*‐dsRNA‐CPP6. (b) Bacterial multiplication rate, (c) lesion length at 10 dps and (d) expression of *SWEET14* in plants sprayed with *sweet14*‐dsRNA and *sweet14*‐dsRNA‐CPP6 compared to water‐ and CPP6‐sprayed plants. (e) Expression of predicted off‐target gene *PCD* at 2, 4, 6 and 10 dps. (f) Prolonged survival of *sweet14*‐dsRNA‐CPP6‐sprayed plants from bacterial infection even after 30 dps, photographs were taken 60 days after germination. Error bars indicate values of means ± *SE* from three biological replicates. The significance of differences was exmined using Student's *t* test (*⍺* = 0.05, *p < 0.05, **p < 0.01, ***p < 0.001, ****p < 0.0001).

Xoo uses the type III secretion system to release TALEs into the host cell, which bind to the promoter of the *Sucrose Will be Eventually Exported Transporter* (*SWEET14*) gene. SWEET14 transporter exports sucrose to the apoplastic region, which favours bacterial survival and multiplication. SWEET14 acts as a negative regulator of plant defence; therefore, transiently silencing it through dsRNA during pathogen infection could protect rice against BLB disease. The 45‐day‐old TN1 rice plants were infected with Xoo then after 24 hpi, *sweet14*‐dsRNA (250 ng/leaf) and *sweet14*‐dsRNA‐CPP6 complexes were sprayed. Bacterial blight disease symptoms at 10 dps were reduced in *sweet14*‐dsRNA‐ and *sweet14*‐dsRNA‐CPP6‐sprayed plants compared to unsprayed control and CPP6‐sprayed plants (Figure 7a). The bacterial growth at 2 dps was reduced >1‐fold in *sweet14*‐dsRNA‐and >2‐fold in *sweet14*‐dsRNA‐CPP6‐sprayed plants (Figure 7b). Lesion length reduced >2‐fold in *sweet14*‐dsRNA‐ and >4‐fold in *sweet14*‐dsRNA‐CPP6‐sprayed plants (Figure 7c). The transcript levels of *SWEET14* remained unchanged at 2 dps in *sweet14*‐dsRNA‐treated plants, whereas it was reduced >1‐fold in *sweet14*‐dsRNA‐CPP6‐sprayed plants. At 4 dps, transcripts were reduced >3‐fold in *sweet14*‐dsRNA‐sprayed plants and >6‐fold in *sweet14*‐dsRNA‐CPP6‐sprayed plants. At 6 dps *SWEET14* transcripts were reduced >9 fold in *sweet14*‐dsRNA‐ and *sweet14*‐dsRNA‐CPP6‐sprayed plants. At 10 dps, *SWEET14* transcripts were reduced >2‐fold in naked *sweet14*‐dsRNA‐ and *sweet14*‐dsRNA‐CPP6‐sprayed plants (Figure 7d). The expression of SWEET14 off‐target predicted gene *Programmed cell death* (*PCD*) was induced in 4 and 6 dps in *sweet14*‐dsRNA‐CPP6‐sprayed plants (Figure 7e). Interestingly, *sweet14*‐dsRNA‐CPP6‐sprayed plants completely recovered after pathogen infection and survived, whereas water‐, CPP6‐ and *sweet14*‐dsRNA‐sprayed plants did not recover (Figure 7f).

## 3 DISCUSSION

We have demonstrated the efficiency of CPP6 as a nanocarrier to deliver dsRNA through foliar spray to induce gene silencing (SIGS), manipulating plant endogenous genes for crop protection and improvement. The success of gene silencing in field conditions can only be achieved by spraying the dsRNA targeting relevant genes. The major challenge in SIGS is dsRNA stability, delivery and systemic spread for target gene silencing (Li et al., 2015). Another challenge in exogenous applications is the cell wall barrier to deliver nucleic acids into plant cells. These challenges forced us to apply nanomaterials in transfecting nucleic acids for crop improvement. In plants, the negative charge of dsRNA prevents passive transport through negatively charged membranes (Wytinck et al., 2020). To deliver siRNAs, abrasion and high‐pressure sprays have been used to improve the cellular uptake in plants (Dalakouras et al., 2016). siRNAs are known to move cell‐to‐cell (short range) and systemically (long range) movement through plasmodesmata and vascular phloem tissue (Melnyk et al., 2011). The delivery of dsRNA is highly dependent on their absorption into cells. Many cationic cell‐penetrating peptides and lipid carriers have been used for gene delivery (Milletti, 2012). Various nanomaterials like carbon nanotubes, magnetic nanoparticles, mesoporous silica nanoparticles and LDH nanoparticles have been reported to deliver nucleic acids into plant cells (Bao et al., 2016; Kolge et al., 2021; Mitter, Worrall, Robinson, Li, et al., 2017). CPP effectively encapsulated the dsRNA at a 1:1 ratio and was stable at various pH and temperatures. CPP6 could systemically spread from the site of infection, as evidenced by NIR signals in distal leaves and *GFP* silencing in transgenic *Arabidopsis* plants. CPP6 was also taken up through roots and spread systemically to the shoots in rice, which could help deliver a wide variety of agronomically important molecules other than nucleic acids. dsRNA can also enter plant cells through the foliar spray and spread systemically to shoots, leaves and roots, probably through phloem. However, the exact mechanism behind the uptake and translocation remains unclear. CPP is highly soluble in water, and simple detergent‐like compounds in plant cells can easily release the conjugated dsRNA effectively. Poly‐aspartic acid is biodegradable (Adelnia et al., 2019) and thus, CPP6 may not pose any environmental threat to use in agricultural fields. The efficient binding of CPPs to nucleic acids can suppress enzymatic degradation. The CPP6 binding to the template RNA inhibited translation, resulting in effective gene silencing.

Large‐scale production and application of dsRNAs and their adaptation in farming could enhance agricultural productivity, reduce malnutrition and sustain food security by manipulating relevant genes. Targeting specific genes may improve basic agronomic traits, grain yield, fruit quality and enhance shelf‐life. The dsRNA‐CPP6 targeting *FT* and *PIF4* delayed flowering, which resulted in more leaves and increased biomass. The reduced expression of predicted off‐target genes *MgT* and *PMEI* is mainly due to a delay in flowering, where signals for floral transition did not induce the expression of these genes. In *Arabidopsis* and rice, overexpression of *miR156* targeting *SQUAMOSA* (*SQUA*) *promoter*‐*binding*‐*like* (*SPL*) gene, delays flowering and increases biomass (Schwab et al., 2005; Xie et al., 2006). The transcription factor PIF4 directly binds to the promoter of *AtFT* and induces flowering (Kumar et al., 2012). Silencing of *SlPIF4* in tomatoes causes stunted growth, 15% reduction in vegetative weight and 23% reduction in fruit weight with 21% total reduction in biomass (Rosado et al., 2019).

The dsRNA applications to modify the plant metabolic process could be very attractive to alter plant growth and development. RNAi hairpin construct can modulate the blue flower colour of *Torenia* into white and pale (Guo et al., 2016). Suppression of *chalcone isomerase* (*CHI*) through RNAi silencing changes flavonoid components in tobacco flowers and reduces pigmentation (Guo et al., 2016). The exogenous application of dsRNA*s* through CPP6 targeting *phytoene desaturase* (*OsPDS1*) leads to stunted growth and yellowing of the rice leaves due to the reduced accumulation of carotenoids. Seedlings attained this phenotype by taking up the *pds*‐dsRNA through roots and CPP6 could be systemically translocated to shoots to induce silencing of the *PDS* gene.

Using dsRNA‐CPP6 to target negative regulators of plant defence genes efficiently protected rice plants from bacterial infection. Plant‐pathogenic bacteria can destroy whole crops if not controlled, they secrete effectors to hijack plant mechanisms to cause virulence (Ramu, Oh, et al., 2021). Many negative regulators are upregulated during pathogen infections along with effector‐hijacked genes (Pal et al., 2022). Much evidence has been reported on possible target genes for virus, insect and fungal resistance (Rank & Koch, 2021); however, no study has reported targeting plant genes to improve bacterial disease resistance. Naked dsRNAs targeting different *CYP51* genes in barley can reduce Fusarium wilt (Koch et al., 2019). dsRNA targeting *sorbitol dehydrogenase* and *phospholipase D* in potatoes reduced the sporulation of *Phytophthora infestans* (Kalyandurg et al., 2021). RNAi‐mediated silencing has been used to improve the host‐defence system in crop plants (Hollomon, 2012). In *Agrobacterium tumefaciens* infection, *iaaM* and *ipt* genes are involved in crown gall disease, and RNAi‐mediated silencing reduces tumour production in *Arabidopsis* (Escobar et al., 2001). In legumes, several miRNA families target *NBS‐LRR* receptors of plant innate immunity in tomatoes and other crops (Shivaprasad et al., 2012).

Targeting *SDIR1*, an E3 ligase that is a negative regulator of plant defence, provided protection against bacterial leaf blight caused by Xoo in rice plants. Targeting *SDIR1* inhibited the bacterial multiplication rate and reduced lesion lengths in dsRNA‐CPP6‐sprayed plants. Induction of *Argonaute* and *Dicer‐like protein 1* indicates activation of the RNAi machinery in *sdir1*‐dsRNA‐sprayed plants. The induction of these genes in dsRNA‐CPP6 is lower because of the slow release of dsRNA in the plant system. The Xoo TALE protein target *SWEET14* plays a negative role in plant defence during bacterial blight disease. CRISPR mutants targeting the promoter of *SWEET14* show improved resistance in rice plants (Oliva et al., 2019). The *SWEET14* off‐target gene *PCD1* showed a higher levels of transcripts, which could also contribute to improved tolerance (Verma et al., 2019). The results suggest that dsRNA with CPP6 does not induce any off‐target gene effects. The transient silencing of *SWEET14* using *sweet14*‐dsRNA‐CPP6 showed reduced bacterial disease symptoms and multiplication rate and enhanced resistance. Even after 30 days *sweet14*‐dsRNA‐CPP6‐sprayed plants showed better tolerance against bacterial disease than dsRNA‐sprayed plants and completely recovered. This suggests that the slow release of dsRNAs by CPP6 in silencing the target gene for a longer duration could provide durable crop protection. Our study demonstrates that the CPP‐based nanoformulations could stabilize different dsRNA constructs and effectively deliver them into plants. Such nanoformulations can also be used to manipulate plant genes involved in stress tolerance and crop protection to attain sustainability.

## 4 EXPERIMENTAL PROCEDURES

### 4.1 Plant growth and bacterial strain

The *A. thaliana* (Col‐0) plants were grown in a growth chamber (22°C and 50%–60% relative humidity) with a 16/8 h light/dark period. The rice TN1 seeds were soaked in water overnight and germinated on filter paper in a Petri plate. Germinated seedlings were transferred to pots and maintained for 45 days in the greenhouse (28°C and 50%–60% relative humidity) with a 16/8 h light/dark photoperiod. The Xoo strain was cultured in nutrient broth (NB) in a shaking incubator at 28°C for 2 days. Nutrient broth was prepared using peptone 5 g, NaCl 5 g, beef extract 1.5 g and yeast extract 1.5 g (HiMedia) in 1 L of distilled water (pH 7–7.2). Nutrient agar (NA) was prepared using peptone 5 g, NaCl 5 g, beef extract 1.5 g, yeast extract 1.5 g and agar powder 15 g (Sigma) in 1 L of distilled water.

### 4.2 Synthesis of nanopolymers

#### 4.2.1 CPP6 nanopolymer

Cationic poly‐aspartic acid‐derived amphiphilic polymers were synthesized using (*N*,*N*‐dimethylaminopropyl)‐polyasapartamide, PADA as described previously (Yavvari et al., 2017).

#### 4.2.2 LDH nanopolymer

MgAl‐LDH polymer was synthesized according to Xu et al. (2006). Briefly, 10 mL of solution containing MgCl_2_ (3 mmol) and AlCl_3_ (1 mmol) was added to 40 mL of 0.15 M NaOH solution under vigorous mixing. Pure LDH slurry was obtained through centrifugation followed by washing and then dispersed in deionized water, and autoclaved at 100°C for 16 h, resulting in homogenous suspension.

### 4.3 NIR labelling and confocal imaging

CPP6 was labelled with Cy7 using a synthetic chemistry approach as described previously (Yavvari et al., 2019). The Cy7‐CPP6 fluorophore was used to test the systemic movement of polymer in plants using NIR imaging. Cy5‐dUTP (650 nm excitation, 670 nm emission) labelled *gfp*‐dsRNA and Cy5‐*gfp*‐dsRNA‐CPP6 were infiltrated to 3‐week‐old *N. benthamiana* plants, at 24 hpi signals were visualized in 40× oil immersion using a confocal laser‐scanning microscope (TCS SP5; Leica). Similarly, Cy5‐*gfp*‐dsRNA and Cy5‐*gfp*‐dsRNA‐CPP6 were added to the tubes, and after 24 h, signals were visualized in rice leaves using confocal microscopy.

### 4.4 Nanopolymer uptake and systemic movement in plants

To test the systemic movement of polymer into plants through infiltration or root uptake, a Cy7‐labelled CPP6 polymer was infiltrated in 3‐week‐old *N. benthamiana* and *A. thaliana* plants. After 24 and 48 h of infiltration, the whole plant was visualized under SPECTRUM In Vivo Imaging System (Perkin Elmer). In rice, the labelled polymer was added in the water, allowed to be taken up through roots and visualized after 24 and 48 h for signals.

### 4.5 Toxicity of nanopolymers on rice growth and development

TN1 seeds were soaked overnight with four different concentrations (5, 25, 50 and 75 μg) of each polymer CPP1, CPP2, CPP4, CPP6 and CPP8. For each concentration, 20 seedlings were used for germination on wet filter paper. Shoot and root length were measured after 4 and 7 days of germination. The seeds grown in distilled water were used as a control.

### 4.6 dsRNA and fluorescent‐labelled dsRNA synthesis

Using the pssRNAit tool (https://www.zhaolab.org/pssRNAit/), a region for dsRNA synthesis having fewer off‐targets and more siRNAs was selected as described (Ahmed et al., 2020) (Table S1). The predicted off‐target genes for each dsRNA were also identified from the pssRNAit tool. The primers were designed for that region and custom synthesized (Table S2). T7 promoter sequences were added to each 5′ end of the DNA template using PCR amplification. DNA template was purified and converted to dsRNA using an in vitro transcription kit (New England Biolabs). Residual DNA was removed from the transcription reaction using DNase I treatment, purified using phenol‐chloroform precipitation and eluted in 30 μL of nuclease‐free water. The dsRNA concentration was quantified using a NanoDrop spectrophotometer (Thermo Fisher). For labelled dsRNA synthesis, 0.5 μL of 10 mM dUTP (uridine triphosphate) labelled with Cy5 was added during the in vitro transcription reaction.

### 4.7 Preparation of dsRNA‐nanoconjugate and biochemical characterization

In vitro‐transcribed dsRNA was incubated with CPP6 at a 1:10 ratio at room temperature for 30 min. We performed dynamic light scattering for biochemical characterization by measuring the hydrodynamic diameter (in nm) to recognize any size variation for up to 25 days for *gfp*‐dsRNA‐CPP6 and 15 days for *pds*‐dsRNA‐CPP6 using Malvern instrument Zetasizer Nanoseries, Nano‐ZS90. ZP (mV) was measured to assess the surface charge/potential of the complex.

### 4.8 Stability and release of dsRNA with nanoparticles in different conditions

The 500 ng of *gfp*‐dsRNA and 5000 ng of CPP6 were conjugated and mixed in tubes and then incubated at 4, 37 and 48°C for 0 min, 2 h, 6 h, 12 h, 24 h, 48 h, 3 days, 4 days, 5 days and 10 days. For pH‐dependent stability, the conjugated *gfp*‐dsRNA‐CPP6 was incubated at pH 7, 5, and 3 for 0 min, 2 h, 6 h, 12 h, 24 h, 48 h, 3 days, 4 days, 5 days and 10 days. The samples were analysed on 2% agarose gel electrophoresis. To assess the RNase‐mediated degradation of *gfp*‐dsRNA‐CPP6 complexes, 1 μL (10 mg/mL) of RNase A (Thermo Fisher) was added to the complexes and incubated at 4, 37 and 48°C for 0, 30 min, 2 h, 6 h, 12 h, 24 h and 48 h and resolved on 2% agarose gel. For the release of dsRNA from the complex, different SDS concentrations were added in a reaction for 30 min and analysed on a 2% agarose gel.

### 4.9 Transcription and translation assay

To check the DNA template accessibility for polymerase activity in the presence of CPP6, 50 ng template was used for PCR amplification, using *GFP*‐specific primers in all reactions. CPP6 and *gfp*‐dsRNA were added at 100 or 200 ng to the individual reactions, the *gfp*‐dsRNA‐CPP6 complex was made and added to the reaction. Similarly, combinations were made with LDH polymer, *gfp*‐dsRNA, kept for 25 and 40 cycles in a thermal cycler and analysed on 0.8% agarose gel. For translation efficiency, dsRNA targeting *luc* was designed. The *luc*‐dsRNA and *luc*‐dsRNA‐CPP6 were used in PURExpress In Vitro Protein Synthesis Kit (New England Biolabs) for assessing the translation.

### 4.10 *Arabidopsis* *GFP* ‐expressing plants

To assess the gene silencing and monitor the expression pattern, *RPL10‐GFP* transgenic plants were generated by the floral dip method. *gfp*‐dsRNA‐CPP6 complex was made by mixing in vitro‐transcribed *gfp*‐dsRNA with CPP6 at a 1:10 ratio. Naked *gfp*‐dsRNA and *gfp*‐dsRNA‐CPP6 were infiltrated into the leaves of 3‐week‐old *Arabidopsis RPL10*‐*GFP‐OE* transgenic plants. GFP fluorescent signals were visualized under confocal microscopy at 1, 24, 48 hpi and leaf samples were collected for gene expression and western blot analysis. To ascertain the levels of GFP, total protein was extracted in extraction buffer (50 mM Tris MES pH 8.0, 50 mM NaCl, 1% vol/vol NP‐40, 0.1% SDS and 1 mM EDTA; HiMedia) containing 1× plant protease inhibitor cocktail (Sigma‐Aldrich) from infiltrated as well as systemic leaves. Immunoblotting with anti‐GFP antibodies (Sigma‐Aldrich) was used to detect RPL10‐GFP (50 kDa) protein. The experimental groups were untreated control, *gfp*‐dsRNA, CPP6 and *gfp*‐dsRNA‐CPP6 complexes. Experiments were performed with three biological replicates.

### 4.11 Targeting *FT* and *PIF4* in *A. thaliana*

Three‐week‐old Col‐0 plants were sprayed with 125 ng/plant *ft*‐dsRNA‐CPP6 and *pif4*‐dsRNA‐CPP6 before bolting initiation. *ft*‐dsRNA‐CPP6 and *pif4*‐dsRNA‐CPP6 were made by mixing in vitro‐transcribed *ft* and *pif4* dsRNA, respectively, with CPP6 in a 1:10 ratio. Plants were sprayed with only water, CPP6 or naked *ft/pif4*‐dsRNA. At 48 hps, the number of bolts initiated was counted for all sprayed plants. After 10 dps, bolting length and the number of leaves were counted in the plants. Leaf samples were collected at 2, 4, 6, 8 and 10 dps and flowers at 10 dps were collected to study gene expression and protein levels. Experiments were performed with 24 plants, and three samples were pooled for each biological replicate.

### 4.12 Targeting rice *PDS* and *OsbZIP23* through root uptake

*pds*‐dsRNA‐CPP6 and *bzip23*‐dsRNA‐CPP6 complexes were made by mixing in vitro‐transcribed *pds*‐dsRNA with CPP6 in a 1:10 ratio (2 μg of dsRNA: 20 μg of CPP6 per seedling). The rice TN1 seeds were soaked overnight and germinated on a Petri plate for 3 days in dark conditions. Germinated seedlings were transferred to microcentrifuge tubes containing water, CPP6, naked *pds*‐dsRNA and *pds*‐dsRNA‐CPP6. Tubes were kept in dark conditions for growth and maintained in a 16/8 h light/dark photoperiod. After 48 h of treatment, samples were collected to study gene expression levels. After 10 days of treatment, seedling height was measured.

For *OsbZIP23* gene silencing, the germinated rice seedlings were treated with 150 mM NaCl for 24 h, followed by *bzip23*‐dsRNA and *bzip23*‐dsRNA‐CPP6. After 48 h of treatment, shoot and root length were measured, and samples were collected for gene expression analysis.

### 4.13 Targeting rice disease susceptibility genes

*sdir1*‐dsRNA‐CPP6 and *sweet14*‐dsRNA‐CPP6 were made by mixing in vitro‐transcribed *sdir1* and *sweet14* dsRNA, respectively, with CPP6 in a 1:10 ratio. The TN1 seeds were soaked in water overnight, germinated on filter paper in a Petri plate and transferred to the soil. After 45 days, leaves were infected with Xoo using the leaf‐clipping method. At 48 hps, leaves were collected, and bacterial multiplication rates were assessed. At 10 dps, photographs were taken, and lesion lengths were measured for bacterial disease symptoms. Leaf samples were collected at 2, 4, 6 and 10 dps for gene expression analysis.

### 4.14 Pathogen infection assay using leaf‐clipping method on rice

TN1 rice plants were grown in a growth chamber for 45 days. Overnight grown bacterial suspension was prepared in MES buffer (10 mM MES, 10 mM MgCl_2_), and leaves were infected with Xoo suspension (10^6^ cfu/mL) by leaf clipping. After 24 h, infected plants were divided into four sets to spray water, CPP6, naked dsRNA or dsRNA‐CPP6 independently. Bacterial multiplication rates were analysed at 48 hps by culturing on NA plates. At 10 dps, greyish to chlorotic symptoms from the top to the edge of the leaves were measured and photographs were taken.

### 4.15 Reverse transcription‐quantitative PCR

Total RNA was isolated using TRIzol reagent (Sigma‐Aldrich) from frozen samples in liquid nitrogen. RNA (1 μg) was converted to cDNA using High‐Capacity cDNA Reverse Transcription Kit (Thermo Fisher). dsRNA targeting and off‐target gene‐specific primers were designed and used for quantitative real‐time PCR. The *Actin* genes from *Arabidopsis* and rice were used as internal controls for normalization. The transcript levels were measured by using SYBR Green (HOT FIREPol Evagreen qPCR Mix) in a quantitative real‐time PCR machine (ABI‐Quant studio 6 Real‐Time PCR system; Thermo Fisher). The expression data was collected and further processed using the 2^−ΔΔ*C*t^ method (Livak & Schmittgen, 2001).

### 4.16 Statistics

Statistical significance between the samples was calculated using Student's *t* test, one‐way analysis of variance (ANOVA, Tukey's multiple comparisons test) or two‐way ANOVA (Tukey's multiple comparisons test). All graphs were prepared in GraphPad Prism 9. All the experiments were repeated a minimum of three times.

## CONFLICT OF INTEREST STATEMENT

The authors declare no competing interest.

## Supporting information


**Figure S1.** Toxicity of cationic poly‐aspartic acid‐derived polymers on rice seedling growth.

**Figure S2.** Stability of dsRNA at different temperatures, pH and RNase A treatment.

**Figure S3.** Expression of *ft*‐dsRNA predicted off‐target gene *Mg transporter* (*MgT*) in *Arabidopsis*.

**Figure S4.** Phenotype of *sdir1*‐dsRNA targeted *Arabidopsis* and expression of *SDIR1* in rice.

**Table S1.** List of dsRNA sequences and their predicted siRNAs.

**Table S2.** List of primers.

## Data Availability

All the additional data related to this manuscript have been given in the supplementary data.
