# Supplementary material for: Exogenous application of nanocarrier‐mediated double‐stranded RNA manipulates physiological traits and defence response against bacterial diseases
Source: Mol Plant Pathol. 2024 Jan 19;25(1):e13417. doi: 10.1111/mpp.13417 (PMC10799200; doi:10.1111/mpp.13417)
Supplement: Supplementary file 1 — Figure S1. Toxicity of cationic poly‐aspartic acid‐derived polymers on rice seedling growth. [file MPP-25-e13417-s003.docx]

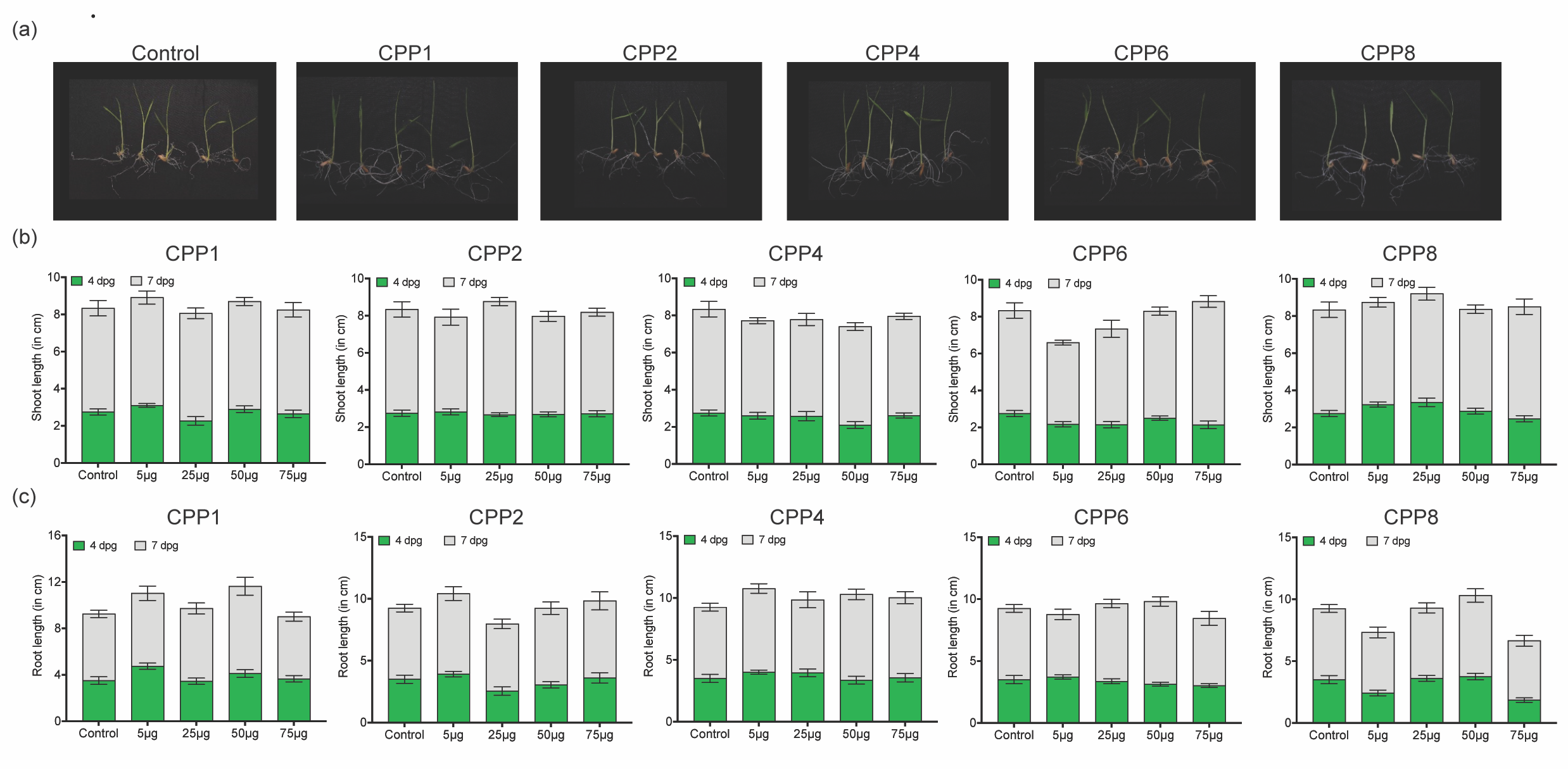


**Figure S1.** **Toxicity of cationic poly-aspartic acid-derived polymers on rice seedling growth,** (a) Phenotype of rice seedlings grown in CPP1, CPP2, CPP4, CPP6 and CPP8 at 75 µg concentration. Photographs were taken after 7 days of post-germination (dpg). (b) Shoot length and, (c) Root length of rice seedlings after 4 and 7 dpg at different concentrations of CPPs. Rice TN1 seeds were soaked in different concentrations of CPPs and allowed to germinate on filter paper in Petri plates for 7 days and shoot and root lengths were recorded at 4 and 7 dpg. Error bars indicate values of means ± SE from a minimum ten biological replicates. The significant difference was determined using One-way ANOVA with Tukeys HSD test (⍺=0.05).
