# Supplementary material for: Exogenous application of nanocarrier‐mediated double‐stranded RNA manipulates physiological traits and defence response against bacterial diseases
Source: Mol Plant Pathol. 2024 Jan 19;25(1):e13417. doi: 10.1111/mpp.13417 (PMC10799200; doi:10.1111/mpp.13417)
Supplement: Supplementary file 2 — Figure S2. Stability of dsRNA at different temperatures, pH and RNase A treatment. [file MPP-25-e13417-s001.docx]

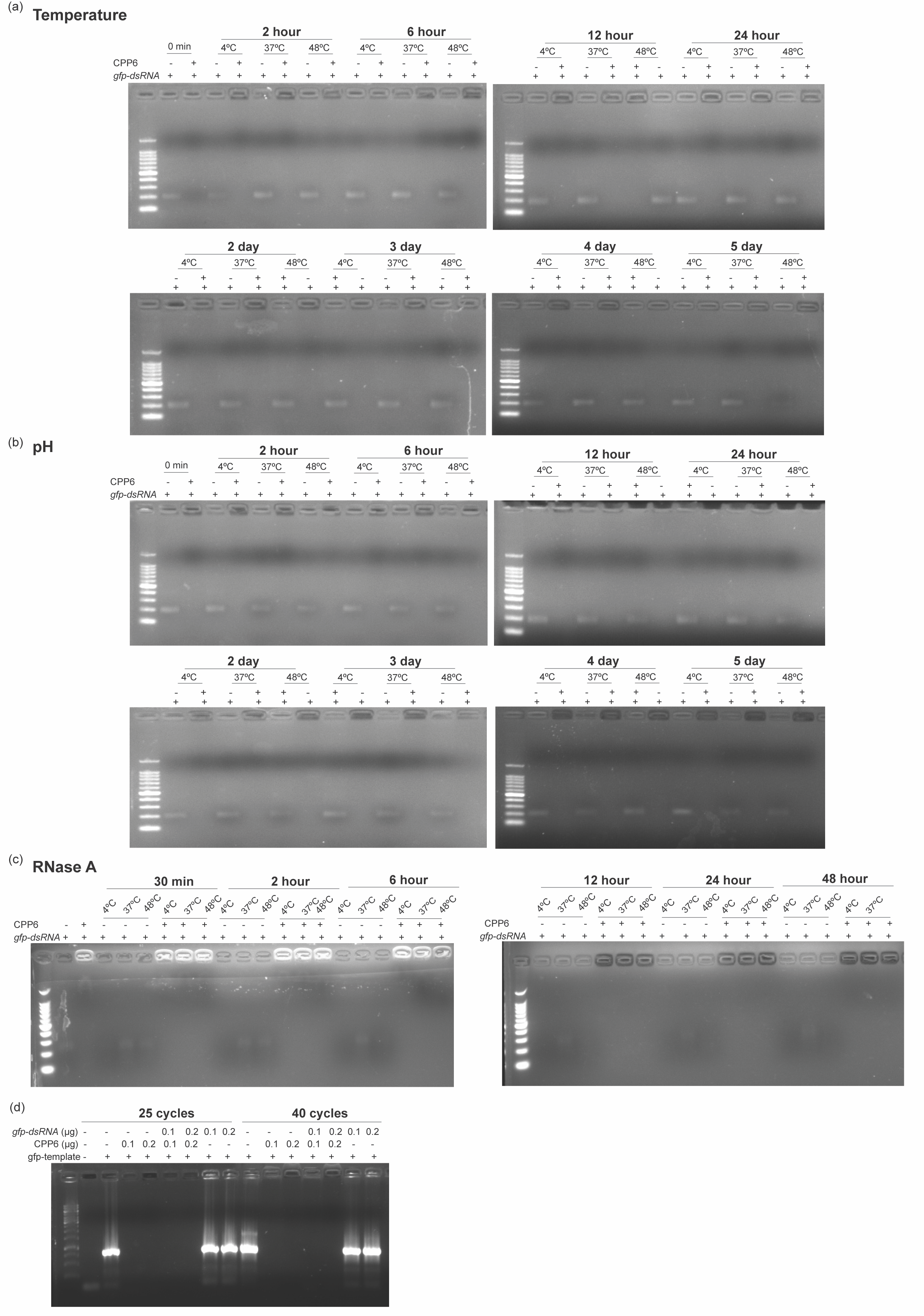


**Figure S2. Stability of dsRNA at different temperatures, pH and RNase treatment:** Stability of dsRNA and *gfp-dsRNA*-CPP6 complexes (a) at different temperatures, b) at different pH and (c) RNase A treatment. Five hundred ng of *gfp-dsRNA* conjugated with 5000 ng of CPP6 and incubated at different temperatures and pH with different time intervals, the complexes were resolved on 2% agarose gel. For RNase treatment, the *gfp-dsRNA-CPP6* complexes were treated with RNase A and incubated at various temperatures for different time intervals. (d) The efficiency of CPP6 in interfering with GFP DNA template accessibility in a PCR reaction with 25 and 40 cycles (94^0^C for 5 min, 94^0^C for 1 min, 59^0^C for 45 sec, 72^0^C for 1 min). 50 ng of GFP DNA template was used in PCR amplification reaction containing different concentrations of CPP6 and *gfp-dsRNA.*
