# Supplementary material for: Exogenous application of nanocarrier‐mediated double‐stranded RNA manipulates physiological traits and defence response against bacterial diseases
Source: Mol Plant Pathol. 2024 Jan 19;25(1):e13417. doi: 10.1111/mpp.13417 (PMC10799200; doi:10.1111/mpp.13417)
Supplement: Supplementary file 3 — Figure S3. Expression of ft‐dsRNA predicted off‐target gene Mg transporter (MgT) in Arabidopsis. [file MPP-25-e13417-s006.docx]

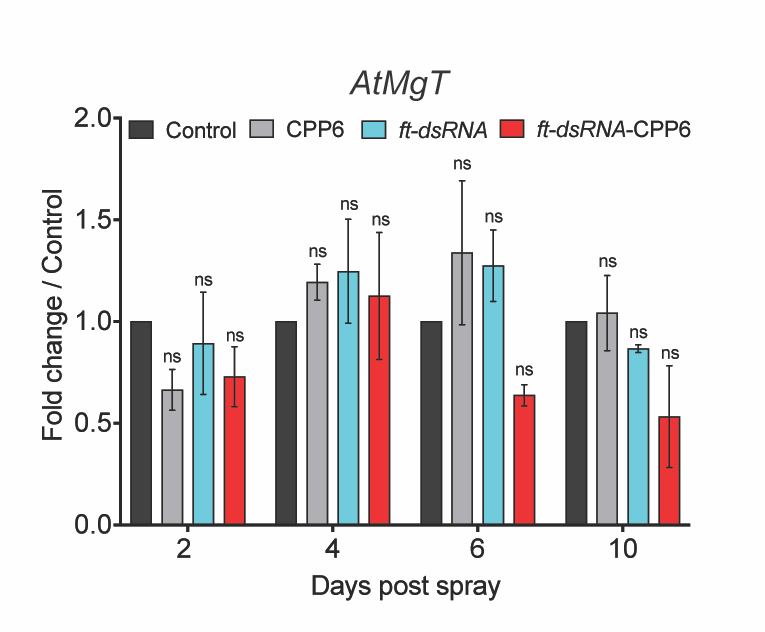


**Figure S3. Expression of *ft-dsRNA* predicted off-target gene Mg transporter (*MgT*) in Arabidopsis.** Three-week-old Arabidopsis plants were sprayed with *ft-dsRNA,* the leaf tissues were frozen at 2, 4, 6 and 10 dps. Total RNA was isolated, converted to cDNA, and used as a template for quantifying *MgT* transcript levels. *AtActin* was used as a normalisation control. Error bars indicate values of means ± SE from three biological replicates. The significant difference was determined using the Student’s T-test (ns-non significant).
