# Supplementary material for: Exogenous application of nanocarrier‐mediated double‐stranded RNA manipulates physiological traits and defence response against bacterial diseases
Source: Mol Plant Pathol. 2024 Jan 19;25(1):e13417. doi: 10.1111/mpp.13417 (PMC10799200; doi:10.1111/mpp.13417)
Supplement: Supplementary file 4 — Figure S4. Phenotype of sdir1‐dsRNA targeted Arabidopsis and expression of SDIR1 in rice. [file MPP-25-e13417-s004.docx]

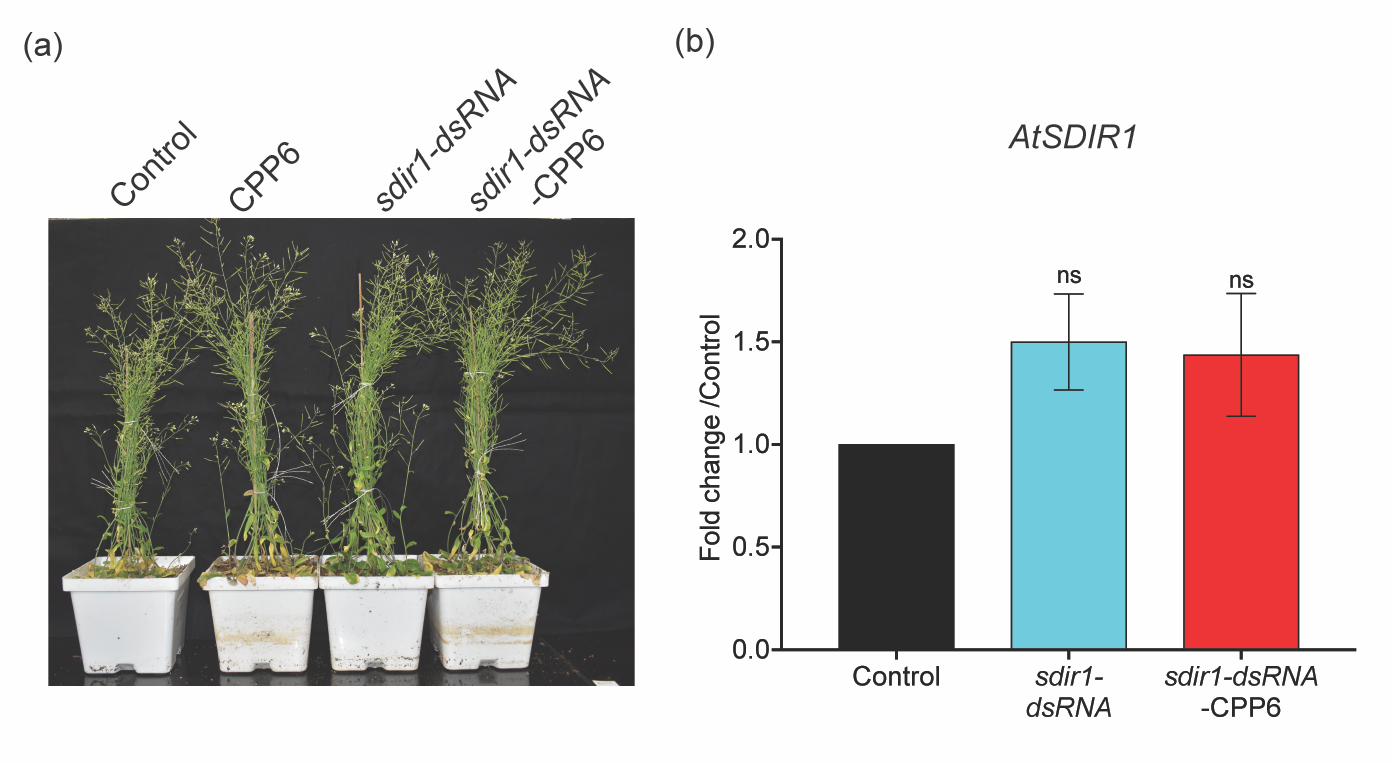


**Figure S4. Phenotype of *sdir1-dsRNA* targeted *Arabidopsis* and expression of *SDIR1* in rice.** (a) CPP6, *sdir1-dsRNA* and *sdir1-dsRNA-*CPP6 were mixed with *Pseudomonas* *syringae* pv. tomato (DC3000) bacterial culture before infiltration, three-week-old Arabidopsis plants were infiltrated, and photographs were taken after 20 dpi. (b) Expression of *SDIR1* gene in rice plants infected with *Xoo, sdir1-dsRNA, sdir1-dsRNA-*CPP6 sprayed plants compared to water sprayed plants. Leaf tissues were collected at 48 hpi and total RNA was converted to cDNA to quantify *SDIR1*. Error bars indicate values of means ± SE from three biological replicates. The significant difference was determined using Student’s T-test (ns-non significant).
