# Supplementary material for: Exogenous application of nanocarrier‐mediated double‐stranded RNA manipulates physiological traits and defence response against bacterial diseases
Source: Mol Plant Pathol. 2024 Jan 19;25(1):e13417. doi: 10.1111/mpp.13417 (PMC10799200; doi:10.1111/mpp.13417)
Supplement: Supplementary file 5 — Table S1. List of dsRNA sequences and their predicted siRNAs. [file MPP-25-e13417-s005.docx]

Supplementary Table S1. List of dsRNA sequences and their predicted siRNAs

| **Gene** | **dsRNA region** | **Predicted siRNAs** |
| --- | --- | --- |
| **GFP**  **(185 bp)** | CTTCTTCAAGGACGACGGGAACTACAAGACACGTGCTGAAGTCAAGTTTGAGGGAGACACCCTCGTCAACAGGATCGAGCTTAAGGGAATCGATTTCAAGGAGGACGGAAACATCCTCGGCCACAAGT TGGAATACAACTACAACTCCCACAACGTATACATCATGGCCGACAAGCAAAAGAACG | CAGUUCAAACUCCCUCUGUGG |
|  |  | UGUCCUAGCUCGAAUUCCCUU |
|  |  | CGGUGUUCAACCUUAUGUUGA |
|  |  | UUGAUGUUGAGGGUGUUGCAU |
|  |  |  |
| ***At*FT**  **(219 bp)** | CTGGAACAACCTTTGGCAATGAGATTGTGTGTTACGAAAATCCAAGTCCCACTGCAGGAATTCATCGTGTCGTGTTTATATTGTTTCGACAGCTTGGCAGGCAAACAGTGTATGCACCAGGGTGGCGCCAGAACTTCAACACTCGCGAGTTTGCTGAGATCTACAATCTCGGCCTTCCCGTGGCCGCAGTTTTCTACAATTGTCAGAGGGAGAGTGGCT | [AACACACAAUCUCAUUGCCAA](file:///pssRNAit/output.do%3fsessionid=1684678423751508&effics=efs&siRNA21=AACACACAAUCUCAUUGCCAA) |
|  |  | [AAUUCCUGCAGUGGGACUUGG](file:///pssRNAit/output.do%3fsessionid=1684678423751508&effics=efs&siRNA21=AAUUCCUGCAGUGGGACUUGG) |
|  |  | [GUUUGCCUGCCAAGCUGUCGA](file:///pssRNAit/output.do%3fsessionid=1684678423751508&effics=efs&siRNA21=GUUUGCCUGCCAAGCUGUCGA) |
|  |  | [UCUGGCGCCACCCUGGUGCAU](file:///pssRNAit/output.do%3fsessionid=1684678423751508&effics=efs&siRNA21=UCUGGCGCCACCCUGGUGCAU) |
|  |  | [UCGCGAGUGUUGAAGUUCUGG](file:///pssRNAit/output.do%3fsessionid=1684678423751508&effics=efs&siRNA21=UCGCGAGUGUUGAAGUUCUGG) |
|  |  | [AGAUCUCAGCAAACUCGCGAG](file:///pssRNAit/output.do%3fsessionid=1684678423751508&effics=efs&siRNA21=AGAUCUCAGCAAACUCGCGAG) |
|  |  | [ACUGCGGCCACGGGAAGGCCG](file:///pssRNAit/output.do%3fsessionid=1684678423751508&effics=efs&siRNA21=ACUGCGGCCACGGGAAGGCCG) |
|  |  | [AAUUGUAGAAAACUGCGGCCA](file:///pssRNAit/output.do%3fsessionid=1684678423751508&effics=efs&siRNA21=AAUUGUAGAAAACUGCGGCCA) |
|  |  |  |
| ***At*PIF4**  **(210 bp)** | TTAATCCGAACGCAAGTTCCTCATCAGGTGGCTCCTCTGGTTGCAGCTTTGGCAAAGATATCAAAGAAATGGCTAGTGGAAGATGCATCACAACCGACCGTAAGAGAAAACGTATAAATCACACTGACGAATCTGTATCTCTATCAGATGCAATCGGTAACAAGTCGAACCAACGATCAGGATCAAACCGAAGGAGTCGAGCAGCTGAAG | [AGGAACUUGCGUUCGGAUUAA](file:///pssRNAit/output.do%3fsessionid=1684678708054377&effics=efs&siRNA21=AGGAACUUGCGUUCGGAUUAA) |
|  |  | [GAUGCAUCUUCCACUAGCCAU](file:///pssRNAit/output.do%3fsessionid=1684678708054377&effics=efs&siRNA21=GAUGCAUCUUCCACUAGCCAU) |
|  |  | [UACAGAUUCGUCAGUGUGAUU](file:///pssRNAit/output.do%3fsessionid=1684678708054377&effics=efs&siRNA21=UACAGAUUCGUCAGUGUGAUU) |
|  |  | [UGUUACCGAUUGCAUCUGAUA](file:///pssRNAit/output.do%3fsessionid=1684678708054377&effics=efs&siRNA21=UGUUACCGAUUGCAUCUGAUA) |
|  |  |  |
| ***Os*PDS**  **(481 bp)** | AAGTATCATGTTGTGAAGACACCAAGGTGAGGACATTTTGCAAGAGCGCCCCCTATCTGATATATCATAGGTAGGTCTAATAGTTGGATGCACACTTCTCTCACGTTCCTTTCTTTTCTGTCTCACTGTTACAGATCTGTTTACAAGACTATCCCGGACTGTGAACCTTGCCGACCTCTGCAAAGATCACCGATTGAAGGGTTCTATCTAGCTGGTGACTACACAAAGCAGAAATATTTGGCTTCGATGGAGGGTGCAGTTCTATCTGGGAAGCTTTGTGCTCAGTCTGTAGTGGAGGTAAACGCTGCTCTCCATGGTTCTGTTTGTACATAGATGCATCAGACTTGTATTGTTGTCTTGGTGCAGTTCACAATGATTCAGTTTTGTAGGCTAATGAGTTATCACTTGCTGATTTCAGGATTATAAAATGCTATCTCGTAGGAGCCTGAAAAGTCTGCAGTCTGAAGTTCCTGTTGCCTCC | [GUCGGCAAGGUUCACAGUCCG](https://www.zhaolab.org/pssRNAit/output.do?sessionid=1684679348089611&effics=efs&siRNA21=GUCGGCAAGGUUCACAGUCCG) |
|  |  | [CUUUGCAGAGGUCGGCAAGGU](https://www.zhaolab.org/pssRNAit/output.do?sessionid=1684679348089611&effics=efs&siRNA21=CUUUGCAGAGGUCGGCAAGGU) |
|  |  | [CAAUCGGUGAUCUUUGCAGAG](https://www.zhaolab.org/pssRNAit/output.do?sessionid=1684679348089611&effics=efs&siRNA21=CAAUCGGUGAUCUUUGCAGAG) |
|  |  | [UAGAACCCUUCAAUCGGUGAU](https://www.zhaolab.org/pssRNAit/output.do?sessionid=1684679348089611&effics=efs&siRNA21=UAGAACCCUUCAAUCGGUGAU) |
|  |  | [UCACCAGCUAGAUAGAACCCU](https://www.zhaolab.org/pssRNAit/output.do?sessionid=1684679348089611&effics=efs&siRNA21=UCACCAGCUAGAUAGAACCCU) |
|  |  | [UGCUUUGUGUAGUCACCAGCU](https://www.zhaolab.org/pssRNAit/output.do?sessionid=1684679348089611&effics=efs&siRNA21=UGCUUUGUGUAGUCACCAGCU) |
|  |  | [CGAAGCCAAAUAUUUCUGCUU](https://www.zhaolab.org/pssRNAit/output.do?sessionid=1684679348089611&effics=efs&siRNA21=CGAAGCCAAAUAUUUCUGCUU) |
|  |  | [UGCACCCUCCAUCGAAGCCAA](https://www.zhaolab.org/pssRNAit/output.do?sessionid=1684679348089611&effics=efs&siRNA21=UGCACCCUCCAUCGAAGCCAA) |
|  |  | [CAGAUAGAACUGCACCCUCCA](https://www.zhaolab.org/pssRNAit/output.do?sessionid=1684679348089611&effics=efs&siRNA21=CAGAUAGAACUGCACCCUCCA) |
|  |  | [CACAAAGCUUCCCAGAUAGAA](https://www.zhaolab.org/pssRNAit/output.do?sessionid=1684679348089611&effics=efs&siRNA21=CACAAAGCUUCCCAGAUAGAA) |
|  |  | [UACAGACUGAGCACAAAGCUU](https://www.zhaolab.org/pssRNAit/output.do?sessionid=1684679348089611&effics=efs&siRNA21=UACAGACUGAGCACAAAGCUU) |
|  |  | [AAUCCUCCACUACAGACUGAG](https://www.zhaolab.org/pssRNAit/output.do?sessionid=1684679348089611&effics=efs&siRNA21=AAUCCUCCACUACAGACUGAG) |
|  |  | [UAGCAUUUUAUAAUCCUCCAC](https://www.zhaolab.org/pssRNAit/output.do?sessionid=1684679348089611&effics=efs&siRNA21=UAGCAUUUUAUAAUCCUCCAC) |
|  |  | [CAGGCUCCUACGAGAUAGCAU](https://www.zhaolab.org/pssRNAit/output.do?sessionid=1684679348089611&effics=efs&siRNA21=CAGGCUCCUACGAGAUAGCAU) |
|  |  | [UGCAGACUUUUCAGGCUCCUA](https://www.zhaolab.org/pssRNAit/output.do?sessionid=1684679348089611&effics=efs&siRNA21=UGCAGACUUUUCAGGCUCCUA) |
|  |  | [AACAGGAACUUCAGACUGCAG](https://www.zhaolab.org/pssRNAit/output.do?sessionid=1684679348089611&effics=efs&siRNA21=AACAGGAACUUCAGACUGCAG) |
|  |  |  |
| ***At*SDIR1 (133 bp)** | AGAACAAGTTACCGTAG GGGAAATCGTTCGCACCTTACCTTGTTTGCATCAGTTTCATGCAGGATGTATCGATCCATGGTTGAGACAGCAAGGAACATGTCCTGTCTGTAAATTTAGAGCTCATTCAGGATGG | AUGGCAUCCCCUUUAGCAAGC |
|  |  | GGCAUCCCCUUUAGCAAGCGU |
|  |  | AUCCCCUUUAGCAAGCGUGGA |
|  |  | UAGCAAGCGUGGAAUGGAACA |
|  |  | AACGUAGUCAAAGUACGUCCU |
|  |  | ACGUAGUCAAAGUACGUCCUA |
|  |  | UGGCAUCCCCUUUAGCAAGCG |
|  |  |  |
| ***Os*SDIR1**  **(179 bp)** | AGGATCGTTTCAAGGCTGATGCAACTGACAACACCCTGGAGGATGAGTTGACATGCAGTGTTTGCTTAGAACAAGTCGTTGTGGGTGATCTATTGAGAAGCCTACCATGCCTGCACCAGTTTCATGCAAACTGCATCGATCCATGGTTGCGCCAACAGGGAACATGCCCAGTTTGCAAG | CUAGCAAAGUUCCGACUACGU |
|  |  | AGCAAAGUUCCGACUACGUUG |
|  |  | UCCGACUACGUUGACUGUUGU |
|  |  | ACGUUGACUGUUGUGGGACCU |
|  |  | ACUGUUGUGGGACCUCCUACU |
|  |  | GUUGUGGGACCUCCUACUCAA |
|  |  | UUGUGGGACCUCCUACUCAAC |
|  |  | GUGGGACCUCCUACUCAACUG |
|  |  | AACUGUACGUCACAAACGAAU |
|  |  | UACGUCACAAACGAAUCUUGU |
|  |  | AACGAAUCUUGUUCAGCAACA |
|  |  | GUUCAGCAACACCCACUAGAU |
|  |  |  |
| ***Os*SWEET14**  **(189 bp)** | CCCCAAGAAGGCCAAGATGTTCACCGCCAAGCTCCTCCTCCTCGTCAACGTCGGCGTCTTCGGCCTCATCCTCCTCCTCACCCTCCTCCTCTCCGCCGGCGACCGCCGCATCGTGGTTCTTGGTTGGGTCTGCGTTGGCTTCTCCGTCAGCGTCTTCGTCGCCCCCCTTAGCATCATCAGGCTGGTGGT | GGGGUUCUUCCGGUUCUACAA |
|  |  | UCCGGUUCUACAAGUGGCGGU |
|  |  | CCGGUUCUACAAGUGGCGGUU |
|  |  | UACAAGUGGCGGUUCGAGGAG |
|  |  | GUGGCGGUUCGAGGAGGAGGA |
|  |  | UGGCGGUUCGAGGAGGAGGAG |
|  |  | GGCGGUUCGAGGAGGAGGAGC |
|  |  | GGUUCGAGGAGGAGGAGCAGU |
|  |  | GAGGAGGAGGAGCAGUUGCAG |
|  |  | GAGCAGUUGCAGCCGCAGAAG |
|  |  | CAGCCGCAGAAGCCGGAGUAG |
|  |  | AGCCGGAGUAGGAGGAGGAGU |
