# Supplementary material for: Exogenous application of nanocarrier‐mediated double‐stranded RNA manipulates physiological traits and defence response against bacterial diseases
Source: Mol Plant Pathol. 2024 Jan 19;25(1):e13417. doi: 10.1111/mpp.13417 (PMC10799200; doi:10.1111/mpp.13417)
Supplement: Supplementary file 6 — Table S2. List of primers. [file MPP-25-e13417-s002.docx]

Supplementary Table 2. List of primers

| **Primer** | **Sequence 5’-3’** | |
| --- | --- | --- |
| IVT_dsRNA-GFP FP | TAATACGACTCACTATAGGGGACCATCTTCTTCAAGGACGA | |
| IVT_dsRNA-GFP RP | TAATACGACTCACTATAGGGTGTTCGTTTTCTTGCCGTAG | |
| IVT_dsRNA AtFT_FP | TAATACGACTCACTATAGGGCTGGAACAACCTTTGGCAAT | |
| IVT_dsRNA AtFT_RP | TAATACGACTCACTATAGGGAGCCACTCTCCCTCTGACAA | |
| IVT_dsRNA AtPIF4_FP | TAATACGACTCACTATAGGGTTAATCCGAACGCAAGTTCC | |
| IVT_dsRNA AtPIF4_RP | TAATACGACTCACTATAGGGCTTCAGCTGCTCGACTCCTT | |
| IVT_dSRNA-PDS FP | TAATACGACTCACTATAGGGGAAGTATCATGTTGTGAAGA | |
| IVT_dsRNA PDS RP | TAATACGACTCACTATAGGG GGAGGCAACAGGAACTTCAG | |
| IVT_dsRNA AtSDIR1 FP | TAATACGACTCACTATAGGGAGAACAAGTTACCGTAG | |
| IVT_dsRNA AtSDIR RP | TAATACGACTCACTATAGGGGCCATCCTGAATGAGC | |
| IVT_dsRNASWEET14 FP | TAATACGACTCACTATAGGGCCCCAAGAAGGCCAA | |
| IVT_dsRNASWEET14 RP | TAATACGACTCACTATAGGGACCACCAGCCTGATG | |
| qRT GFP-FP | TACGTGCAGGAGAGGACCAT | |
| qRT GFP-RP | ACTTGTGGCGCAGGATGTTT | |
| AtFT qRT_FP | CAGGAATTCATCGTGTCGTG | |
| AtFT qRT_RP | AGCCACTCTCCCTCTGACAA | |
| AtPIF4 qRT_FP | ATCGGGGATTAGGGAAACAG | |
| AtPIF4 qRT_RP | TGCGTTCGGATTAAGCTTTT | |
| qRT PDS-FP | GGAGTTGGTCTTTGCTCCTG | |
| qRT PDS-RP | CAAGGTTCACAGTCCGGGAT | |
| AtSDIR1 qRT-FP | GACTTAGATTATGAAACTTT | |
| AtSDIR1 qRT-RP | TCAGCTGAGGATGAGGTTG | |
| qRT_OsSDIR_FP | CAATAGCCCTCATGCTCCTT | |
| qRT_OsSDIR_RP | AAAGAGGCACTTCCCTGATG | |
| OsSWEET14 qRT_FP | ATCGCCGTCTACCTCGTCTA | |
| OsSWEET14 qRT_RP | CACCAGCCTGATGATGCTAA | |
| OsAGO12_qRT_FP | GCATCACACCCGCCTATT | |
| OsAGO12_qRT_RP | CTCGGATGGCAGATCATTGT | |
| OsDicer1_qRT_FP | ACTGATGCCTGGAACAATCC | |
| OsDicer1_qRT_RP | CAAACCCGTATTCCCTCCTATC | |
| AtFTofftarget: MgT_qRT_FP | | TCGAGGGTTCGTCGGTAATA |
| AtFTofftarget: MgT_qRT_RP | | TTGGCGTCTGAGTGGAATG |
| AtFTofftarget: PMeI _qRT_FP | | GAGTCATGCGTGGCTAGTTAT |
| AtFT offtarget: PMeI_qRT_RP | | CAAGTGCTTGCGTGAAAGAG |
| AtSDIR1 offtarget: DEK _qRT_FP | | GTCGAGCTGTCGCATAGAAA |
| AtSDIR1offtarget: DEK_qRT_RP | | CCTCAGCTTCTTCCTCTTCATC |
| AtSDIR1offtarget: RPS12_qRT_FP | | TGCTGGATTTGGTCGTAAGG |
| AtSDIR1offtarget: RPS12_qRT_RP | | GAAGAGGGCCAAGAGTGAAA |
| OsSWEET14offtarget: PCD _qRT_FP | | CCAGAGACTCCCATTGACTTAC |
| OsSWEET14offtarget: PCD_qRT_RP | | CCCTTCCTAACTGTCCATCTTT |
